# Safety and efficacy of anti-IL-5 monoclonal antibodies as second-line therapy for chronic rhinosinusitis with nasal polyps: a meta-analysis

**DOI:** 10.3389/fimmu.2026.1746573

**Published:** 2026-03-30

**Authors:** Huixiu Liao, Chaohong Qiu, Yingying Luo, Jiaru Chen, Congyu Qin, Jieru Quan, Weiming Liang, Jiongying Qin, Rui Chen

**Affiliations:** 1 The First Affiliated Hospital of Guangxi University of Science and Technology, Guangxi University of Science and Technology, Liuzhou, Guangxi, China; 2 Department of Otolaryngology, Rongshui Miao Autonomous County People’s Hospital, Liuzhou, Guangxi, China; 3 School of Economics and Management, Guangxi University of Science and Technology, Liuzhou, Guangxi, China

**Keywords:** anti-IL-5 monoclonal antibodies, chronic rhinosinusitis with nasal polyps, meta-analysis, nasal polyp score, second-line therapy

## Abstract

**Introduction:**

This meta-analysis aimed to evaluate the safety and efficacy of anti-IL-5 monoclonal antibody (anti-IL-5 mAb) as a second-line therapy for chronic rhinosinusitis with nasal polyps (CRSwNP).

**Materials and methods:**

Four databases (PubMed, Web of Science, Embase and Cochrane Library) were searched from the establishment of the databases to September 15, 2025, for randomized controlled trials (RCTs) comparing anti-IL-5 mAb versus placebo in the treatment of CRSwNP. The primary outcome measure of this meta-analysis were nasal polyp score (NPS) and Sino-Nasal Outcome Test-22 (SNOT-22) score. Secondary outcome measures included nasal blockage score (NBS), loss of smell score (LSS), Lund-Mackay score (LMS), University of Pennsylvania Smell Identification Test (UPSIT) score, nasal overall visual analog scale(VAS) score, nasal composite visual analog scale(VAS) score, number of first-time NP surgery patients and number of systemic corticosteroids (≥1 course) patients.

**Results:**

Totally 10 RCTs were included for meta-analysis. Compared with placebo, anti-IL-5 mAb provided a significantly lower NPS (WMD = -0.58, 95%CI: -0.70 ~ -0.46, P < 0.00001, I^2^ = 83%), SNOT-22 score (WMD: -9.57, 95% CI: -11.03 ~ -8.11, P < 0.00001, I^2^ = 80%), NBS (SMD: -1.75; 95% CI: -3.13 ~ -0.36, P = 0.01, I^2^ = 99%), LSS (SMD: -1.68; 95% CI: -3.13 ~ -0.24, P = 0.02, I^2^ = 99%), LMS (WMD: -2.00; 95% CI: -2.65 ~ -1.34, P < 0.00001, I^2^ = 97%), nasal overall VAS score (WMD: -1.54, 95% CI: -1.64 ~ -1.43, P < 0.00001, I^2^ = 0%), nasal composite VAS score (WMD: -1.28, 95% CI: -1.66 ~ -0.19, P < 0.00001, I^2^ = 0%), number of first-time NP surgery patients (RR: 0.64, 95% CI: 0.41 ~ 1.00, P = 0.05, I^2^ = 69%) and number of systemic corticosteroids (≥1 course) patients (RR: 0.73, 95% CI: 0.62 ~ 0.86, P = 0.0002, I^2^ = 0%), while significantly improved UPSIT (WMD: 2.09, 95% CI: 0.42 ~ 3.77, P = 0.01, I^2^ = 0%).

**Conclusions:**

This results confirmed that anti-IL-5 mAb therapy was safe and effective for CRSwNP and can serve as a second-line therapy.

**Systematic review registration:**

https://www.crd.york.ac.uk/prospero/, identifier CRD420251170717.

## 1 Introduction

CRS is estimated to affect 5–15% of the general population, making it one of the most prevalent chronic diseases worldwide (1). According to the EPOS 2020 guidelines, CRS is defined as inflammation of the nasal and paranasal sinuses characterized by two or more symptoms, one of which must be either nasal blockage/obstruction/congestion or nasal discharge (anterior/posterior nasal drip), ± facial pain/pressure, ± reduction or loss of smell, for at least 12 weeks (2). Even though the EPOS 2020 guidelines (2) have updated the classification of CRS, it has traditionally been divided into two primary phenotypes: CRSwNP and chronic rhinosinusitis without nasal polyps (CRSsNP) (3). The pathogenesis of CRS is multifactorial and incompletely understood, involving risk factors: genetic predispositions, environmental triggers (e.g., allergens, air pollutants), and other inflammatory diseases (4). CRS significantly impacts quality of life, particularly CRSwNP. Nasal polyps impact numerous aspects of quality of life—including physical health, overall health, social functioning, sleep, mental health, and workplace absenteeism—due to symptoms arising from nasal obstruction, olfactory impairment, rhinorrhea, and lower respiratory involvement (5, 6). Notably, CRSwNP often coexists with respiratory conditions like asthma and allergic rhinitis, further exacerbating its clinical complexity and worsening patient outcomes, which represents a major healthcare burden, in terms of both direct medical costs and productivity losses (7).

Treatment options for CRSwNP patients are limited. Topical corticosteroids and nasal saline irrigation are both recommended as initial drug treatments (8). Topical corticosteroids effectively reduce polyp size, alleviate postoperative nasal symptoms, and lowered the recurrence rate following polypectomy (9). Additionally, oral corticosteroids are strongly recommended for the short-term treatment of CRSwNP (10). However, some CRSwNP patients exhibited insensitivity to corticosteroid therapy, leading to corticosteroid resistance (2). According to EPOS 2020 and EUFOREA consensus, second-line therapy is indicated after failure of maximal medical therapy (intranasal corticosteroids plus at least one short course of systemic corticosteroids) and/or endoscopic sinus surgery (11, 12). Second-line options include biologics (e.g., dupilumab, omalizumab, mepolizumab) and, in selected cases, aspirin therapy after desensitization (13–16). Among biologics, those targeting type 2 inflammation are particularly relevant because CRSwNP is often characterized by eosinophilic infiltration driven by interleukin (IL)-5 (17, 18).

IL-5 is the key cytokine for eosinophil differentiation, activation, and survival (19). The IL-5 receptor (IL-5R) is expressed on eosinophils, basophils, and their progenitors (20, 21). IL-5Rα exists as a heterodimer and is regarded as the most crucial cytokine receptor expressed on eosinophils (22, 23). Monoclonal antibodies against IL-5 or its receptor have been developed for eosinophilic diseases (23). The anti-IL-5 mAb currently used in clinical trials for the treatment of CRSwNP mainly include benralizumab (anti-IL-5Rα mAb) (24–27), mepolizumab (anti-IL-5 mAb) (28–31), reslizumab (anti-IL-5 mAb) (32) and depemokimab (anti-IL-5 mAb) (33). There are still many others under exploration.

However, the efficacy and safety of anti-IL-5 mAb in the treatment of CRSwNP are still controversial. Therefore, it is necessary to further integrate the existing evidence to clarify the efficacy and safety of anti-IL-5 mAb in CRSwNP. To this end, this meta-analysis was conducted to provide evidence-based support for personalized treatment strategies in CRSwNP by more clearly elucidating the differences in efficacy and safety of anti-IL-5 mAb therapy.

## 2 Materials and methods

### 2.1 Case inclusion and exclusion criteria

#### 2.1.1 Search strategy

The present meta-analysis was conducted in accordance with the 2020 standards of the Preferred Reporting Project for Systematic Review and Meta-Analysis (PRISMA). This meta-analysis had been officially recorded at PROSPERO under the registration number CRD420251170717. A comprehensive search was performed across four databases: PubMed, Web of Science, Embase, and the Cochrane Library, to discover literature published until September 15, 2025. The search strategy included a blend of MeSH and free-text terms in accordance with the PICOS principle. The search keyword was “sinusitis” AND “anti-IL-5 monoclonal antibody” AND “trial”. **Table 1** provided a comprehensive listing of the search results. Furthermore, We also evaluated the reference lists of the relevant studies to identify additional studies. We performed a comprehensive manual evaluation of the bibliographies of the identified articles, together with relevant reviews and meta-analyses, to identify additional new RCTs that satisfied the inclusion criteria.

**Table 1:** Basic characteristics of included trials.

Author, year	Country	Study design	Registration ID	Population	Intervention	Comparison	Cases		NPS, mean (SD)		SNOT-22 score, mean (SD)		Age, years, mean (SD)		Male, n (%)	
							Intervention	Placebo	Intervention	Placebo	Intervention	Placebo	Intervention	Placebo	Intervention	Placebo
Bachert 2022	Europe, USA	RCT	NCT03401229	CRSwNP	Benralizumab 30 mg SC every 8 weeks (first 3 Doses q4w)	Placebo	207	203	6.15 (1.19)	6.13 (1.13)	69.3 (19.77)	69.0 (19.03)	50.1 (12.4)	50.2 (13.9)	142 (68.6)	121 (59.6)
Han 2021	Argentina, Australia, Canada, Germany, Netherlands, South Korea, Romania, Russia, Sweden, UK, USA	RCT	NCT03085797	CRSwNP	Mepolizumab 100 mg SC q4w	Placebo	206	201	5.4 (1.2)	5.6 (1.4)	63.7 (17.6)	64.4 (19.0)	48.6 (13.6)	48.9 (12.5)	139 (67)	125 (62)
Bachert 2017	Belgium, Netherlands, UK	RCT	NCT01362244	CRSwNP	Mepolizumab 750 mg IV q4w	Placebo	54	51	6.28 (0.88)	6.31 (0.88)	51.5 (17.0)	49.5 (19.0)	51 (11)	50 (10)	41 (76)	34 (67)
Fujieda 2024	Japan, Russia, China	RCT	NCT04607005	CRSwNP	Mepolizumab 100 mg SC q4w	Placebo	80	83	5.9 (1.27)	6.1 (1.25)	56.9 (18.94)	55.6 (20.22)	53 (10.7)	52 (13.2)	51 (64)	54 (65)
Gevaert 2006	Belgium, Austria	RCT	NA	CRSwNP	Reslizumab 1 mg/kg IV	Placebo	8	4	6.00 (4.5-7.0)*	6.00 (4.5-8.0)*	NA	NA	43.6 (22-63) *	48 (21-59) *	6 (75)	6 (75)
Gevaert 2011	Belgium	RCT	NA	CRSwNP	Mepolizumab 750 mg IV q4w	Placebo	20	10	5.2 (1.74)	5.5 (1.65)	NA	NA	50.05 (8.86)	45.9 (11.43)	14 (70)	8 (80)
Takabayashi 2021	Japan	RCT	NCT02772419	CRSwNP	Benralizumab 30 mg SC q4w	Placebo	22	5	15.8 (3.8)	16.9 (4.2)	31.7 (18.8)	30.5 (15.8)	53 (12.3)	53.3 (14.8)	12 (52.2)	7 (63.6)
Tversky 2021	USA	RCT	NCT03450083	CRSwNP	Benralizumab 30 mg SC	Placebo	12	12	5.7 (0.87)	6.2 (0.97)	57.6 (16.8)	64.8 (18.3)	49.8 (12.1)	50.8 (13.1)	7 (58)	7 (58)
Canonica 2022	221 clinical research centers worldwide	RCT	NCT03170271	CRSwNP	Benralizumab 30 mg SC every 8 weeks (first 3 doses q4w)	Placebo	96	57	NA	NA	51.5 (20.4)	48.2 (21.2)	53.1 (12.3)	52.6 (11.1)	43 (44.8)	33 (57.9)
Gevaert 2025	Argentina, Belgium, Canada, China, France, Germany, taly, Japan, Netherlands, Poland, Romania, Spain, Sweden, Türkiye, UK, USA	RCT	NCT05274750, NCT05281523	CRSwNP	Depemokimab 100 mg SC	Placebo	272	256	5.9 (1.27)	5.9 (1.37)	59.1 (22.07)	58.3 (20.12)	52.4 (13.27)	51.6 (13.27)	187 (69)	178 (70)

SD, standard deviation. *Mean (interquartile range).

#### 2.1.2 Inclusion and exclusion criteria

The criteria for inclusion were as follows: (1) Patients diagnosed with CRSwNP who had demonstrated poor response to standard therapy;(2) Patients in the intervention group received anti-IL-5 therapy; (3) Patients in the control group received placebo; (4) At least one of the following outcomes was reported: NPS, SNOT-22 score, NBS, LSS, LMS, UPSIT score, nasal overall VAS score, nasal composite VAS score, number of first-time NP surgery patients, number of systemic corticosteroids (≥1 course) patients, adverse events (AEs), any on-treatment AE (≥1 AE) and serious adverse events (SAEs); (5) Study design: randomized controlled trial (RCT).

The criteria for exclusion were as follows: (1) Different types of articles, such as case reports, publications, letters, reviews, meta-analyses, editorials, animal studies, protocols, conferences, etc.; (2) Research on other diseases such as tumors; (3) Trials involving interventions that combine other drugs or treatment regimens; (4) Not relevant; (5) Duplicate patient cohort; (6) The full text cannot be obtained; (7) Data cannot be obtained.

### 2.2 Selection process

The literature selection process, which included the elimination of duplicate entries, was executed with EndNote (Version X7.8; Clarivate Analytics). Two independent reviewers conducted the preliminary search. The redundant items were removed, and the titles and abstracts were evaluated to determine their relevance. The full-text articles were then obtained and assessed by the same reviewers to determine whether they met the inclusion criteria for this meta-analysis. Each study was classified as either included or omitted. We addressed the issue by achieving a consensus. If the parties could not reach an agreement, a third reviewer assumed the position of mediator.

### 2.3 Data extraction

Two independent reviewers extracted data. The extracted data included: (1) Basic characteristics of included studies: authors, nationality, publication year, etc.; (2) Study methods: type of study design, randomization method, allocation concealment, use of blinding, duration, presence of other bias risks, etc.; (3) Baseline characteristics of included subjects: age, sample size, sinus tract status, etc.; (4) Intervention measures: Specific methods for intervention groups and experimental groups, including administration route, dosage, treatment duration, follow-up period, and presence of other interventions; (5) Primary outcome measure: NPS and SNOT-22 score; Secondary outcome measures: NBS, LSS, LMS, UPSIT score, nasal overall VAS score, nasal composite VAS score, number of first-time NP surgery patients and number of systemic (≥1 course) patients; (6) Literature quality assessment scores.

All NPS were total NPS in this meta-analysis, which is determined from bilateral nasal endoscopy and rated on a scale of 0 to 4 for each nostril (total score range, 0-8). Rating criteria: Grade 0 indicated no polyps, Grade 1: Small polyps not reaching the inferior margin of the middle turbinate in the middle nasal meatus; Grade 2: Polyps reaching below the inferior margin of the middle turbinate; Grade 3: Large polyps extending from the inferior turbinate or polyp margins toward the medial aspect of the middle turbinate; Grade 4: Large polyps causing complete obstruction of the inferior nasal meatus.

The SNOT-22 is a 22-item disease-specific health-related quality of life (HRQoL) measure with a total score range from 0 (highest quality of life) to 110 (worst quality of life). Each of the 22 items is scored using a 6-point rating scale ranging from 0 to 5 (0 =Not present/no problem; 1 =Very mild problem; 2 = Mild or slight problem; 3 = Moderate problem; 4 = Severe problem; 5=Problem as “bad as it can be”).

The NBS, the LSS, the nasal overall VAS score and the nasal composite VAS score are assessed using a VAS. Their score ranges from 0 (none) to 10 (worst), except for the assessment by Bachert et al (24) and Gevaert et al (33), which used 0–3 score.

The LMS evaluates the patency using a 0–2 scale (0-normal; 1-partial opacification; and 2-total opacification) of each sinus (maxillary, anterior ethmoid, posterior ethmoid, sphenoid, frontal sinus on each side). The osteomeatal complex is graded as 0- not occluded or 2-occluded. The total LMS is the sum of the scores for all paranasal sinuses on both sides, and ranges from 0 to 24.

The UPSIT is a quantitative test of olfactory function that uses four booklets of 10 questions each on odor identification, with scores based on the number of correctly identified odors (scores range from 0 to 40).

### 2.4 Assessment of the risk of bias

In the present meta-analysis, Two reviewers independently assessed the risk of bias of included trials using the Cochrane Collaboration Risk of Bias (RoB) 2.0 tool for randomized trials. This study evaluated five domains: randomization process, deviations from intended interventions, missing outcome data, measurement of the outcome, and selection of the reported result. Each domain was rated as “low risk,” “some concerns,” or “high risk,” and an overall judgment was derived. The quality assessment was performed by two reviewers, with any disparities addressed by conversation with a third senior author.

### 2.5 Statistical analysis

The statistical analysis was performed with the Review Manager v5.3 software. Continuous data were expressed as mean difference (MD) or standardized mean difference (SMD) with 95% confidence intervals (CI) as effect measures. Heterogeneity was assessed in all meta-analyses using the Cochrane Q p-value and the I2 statistic. Pooled data were analyzed using a fixed-effect model (FEM) when heterogeneity was low or moderate (I^2^ < 50%), and a random-effect model (REM) when heterogeneity was large (I^2^ ≥ 50%). Statistical heterogeneity was evaluated using a standard chi-square test, with significance set at P<0.05. The possible publishing bias was assessed through visual examination of the funnel plots.

## 3 Results

### 3.1 Search results

**Figure 1:**
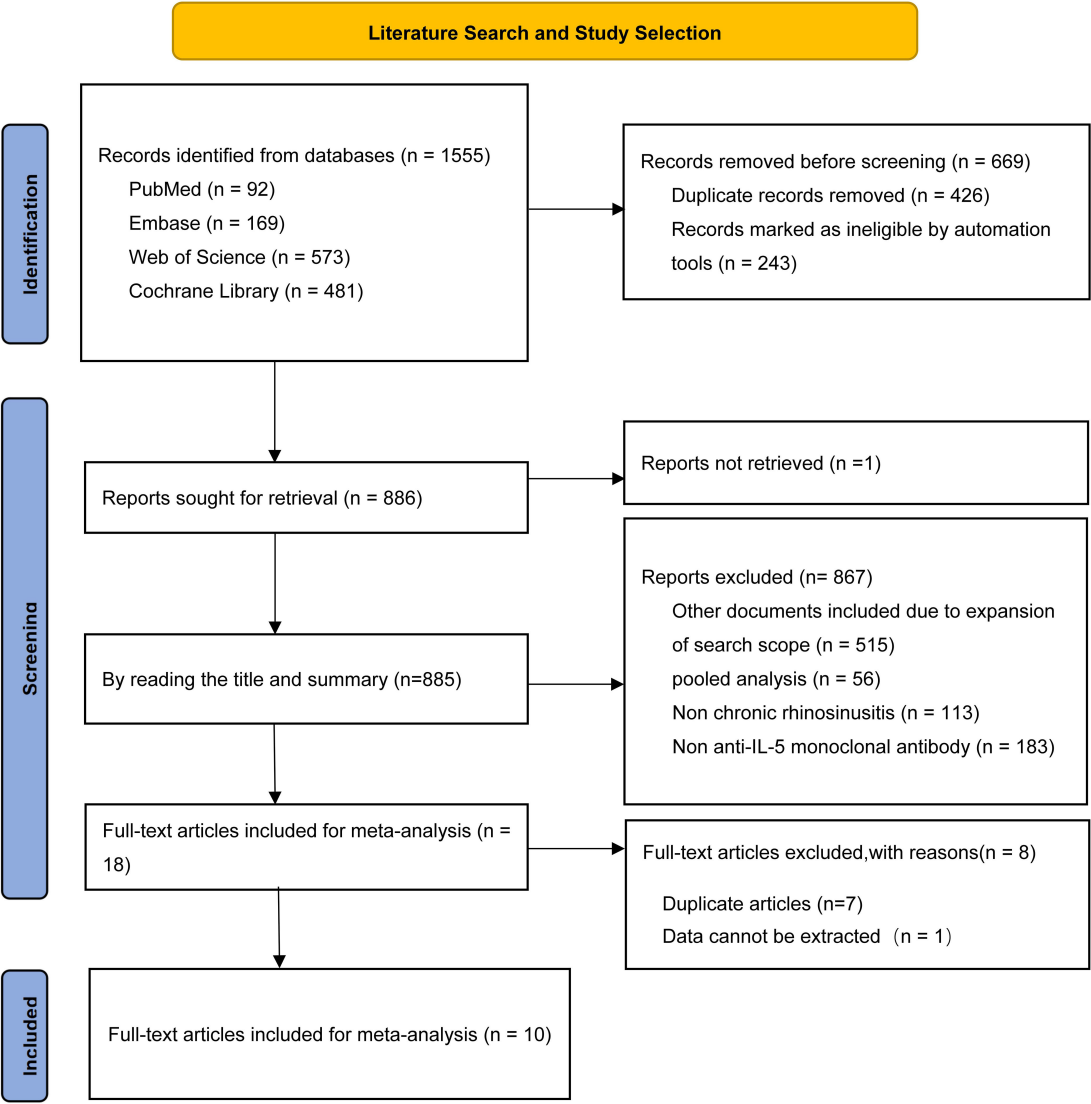
Flow chart of literature search strategies.

This study acquired a total of 1,555 records from four databases. First, duplicate records were screened using the literature management software Endnote, resulting in the exclusion of 426 articles. Records marked as ineligible by automation tools (including literature types such as conference abstracts, editorial material, trial registry records, letters, and reviews) were removed, totaling 243 articles. One Reports not retrieved was also excluded. By reading the titles and summaries, 867 records were excluded for non-anti-IL-5 therapy or non-CRS disease. Full-text review further excluded 8 duplicates or trials with unobtainable data. Ultimately, 10 RCTs (24–33) were incorporated into the final meta-analysis, adhering to the predetermined inclusion and exclusion criteria. The literature screening process was illustrated in **Figure 1**.

### 3.2 Research characteristics

This study included 10 RCTs involving 1,859 patients diagnosed with CRS. Among them, 977 patients received anti-IL-5 monoclonal antibody therapy, while 882 patients received placebo. **Table 1** listed the basic study characteristics of the 10 RCTs (registration ID, authors, year, sinuses, patients, age, outcome measures, etc.). These ten RCTs evaluated anti-IL-5 monoclonal antibody therapy in CRS patients. All patients in the treatment group had CRSwNP. Among these, one trial (31) was a single-center RCT, seven trials (24, 25, 27–30, 33) were multicenter RCTs, and two trials (26, 32) were two-center RCTs. Additionally, the 10 RCTs included in this meta-analysis encompassed four types of anti-5-IL monoclonal antibodies: benralizumab (24–27), mepolizumab (28–31), reslizumab (32), depemokimab (33).

### 3.3 Quality assessment

**Figure 2:**
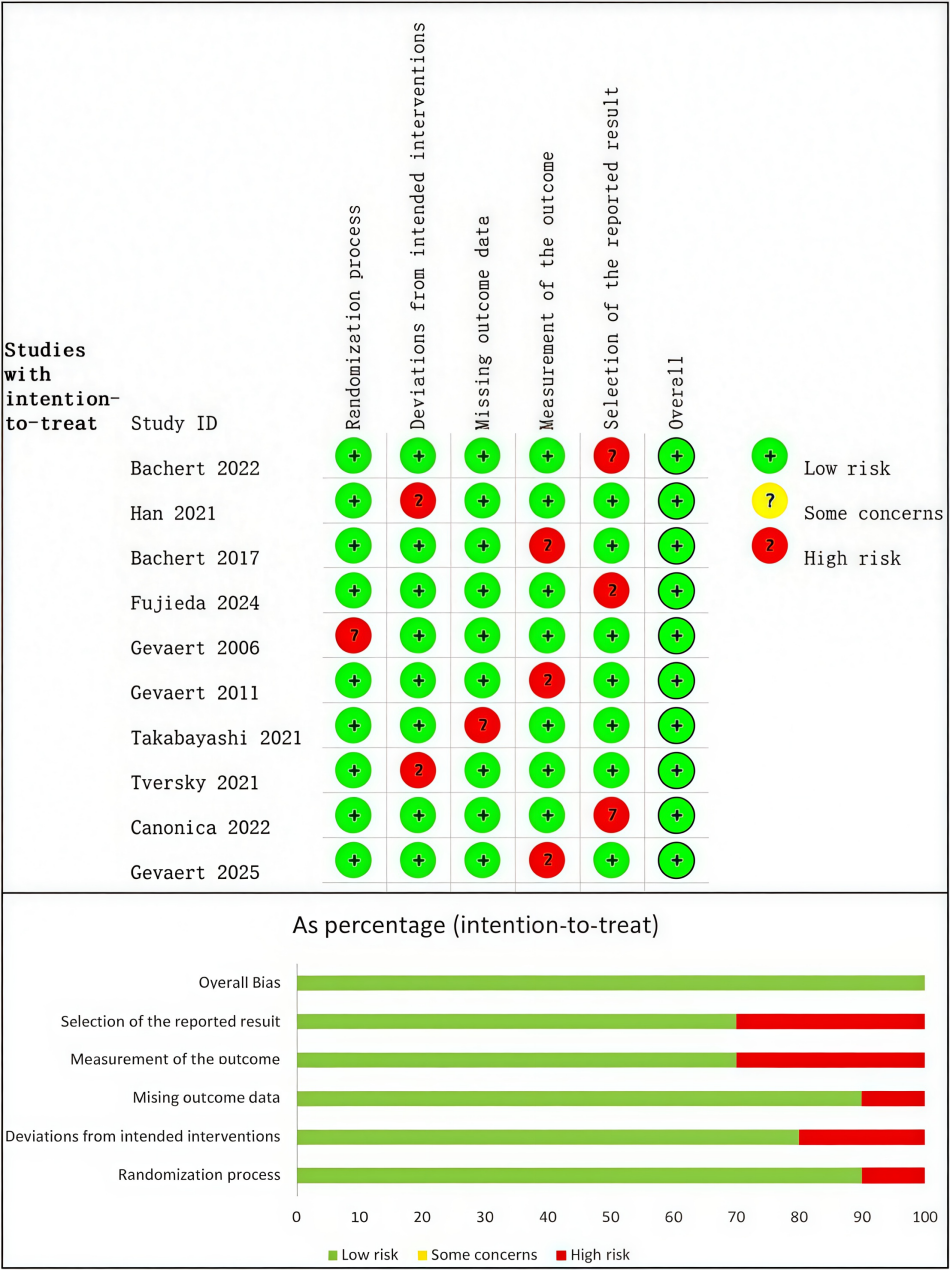
Risk of bias assessment diagram.

The Cochrane Collaboration RoB 2.0 tool was used to assess the risk of bias in the included studies. **Figure 2** provided a brief overview of the risk of bias assessment results. In this study, 10 trials were judged as having a low overall risk of bias. In the randomization process, deviations from intended interventions, missing outcome data, measurement of the outcome, and selection of the reported result, trials were rated as having high risk in these five aspects with 1, 2, 1, 3, and 3 trials respectively.

### 3.4 Clinical outcomes

The meta-analysis results for all outcome measures were summarized in **Table 2**.

**Table 2:** Results of outcome measures.

Outcomes	No. of studies	Sample size		Heterogeneity		Overall effect size	95% CI of overall effect	P value
		Experimental	Control	I^2^(%)	P value			
NPS	9	799	753	83	<0.00001	WMD = -0.58	-0.70 ~ -0.46	<0.00001
SNOT-22 score	8	886	769	80	<0.0001	WMD = -9.57	-11.03 ~ -8.11	<0.00001
NBS	6	772	722	99	<0.00001	SMD = -1.75	-3.13 ~ -0.36	0.01
LSS	6	772	722	99	<0.00001	SMD = -1.68	-3.13 ~ -0.24	0.02
LMS	5	443	414	97	<0.00001	WMD = -2.00	-2.65 ~ -1.34	<0.00001
UPSIT score	2	66	66	0	0.64	WMD = 2.09	0.42 ~ 3.77	0.01
nasal overall VAS score	2	286	284	0	0.41	WMD = -1.54	-1.64 ~ -1.43	<0.00001
nasal composite VAS score	2	286	284	0	0.15	WMD = -1.28	-1.66 ~ -0.19	<0.00001
number of first-time NP surgery patients	3	685	660	69	0.04	RR = 0.64	0.41 ~ 1.00	0.05
number of systemic corticosteroids (≥1 course) patients	3	685	660	0	0.86	RR = 0.73	0.62 ~ 0.86	0.0002

#### 3.4.1 Nasal polyp score

A total of 9 RCTs were included (24, 25, 27–33). The anti-IL-5 therapies were significantly higher improvement versus placebo in the NPS (WMD = -0.58, 95%CI: -0.70 ~ -0.46, P < 0.00001, I^2^ = 83%) (**Supplementary Figure 1**). The results of meta-analysis were not prone to change significantly due to the change of the number of studies and were robust (**Supplementary Figure 2**). An evaluation of publication bias was conducted using funnel plots in relation to TPS (**Supplementary Figure 3**) and the bilaterally symmetrical funnel plot showed no significant evidence of publication bias.

The results of subgroup analysis showed that benralizumab (WMD = -0.55, 95%CI: -0.73 ~ -0.38, P < 0.00001, I^2^ = 0%), mepolizumab (WMD = -0.77, 95%CI: -1.16 ~ -0.39, P < 0.00001, I^2^ = 75%) and depemokimab (WMD = -0.60, 95%CI: -0.62 ~ -0.58, P < 0.00001) were significant improvement in NPS compared with placebo, but not reslizumab (WMD = -0.75, 95%CI: -2.54 ~ 1.04, P = 0.41, I^2^ = 0%). (**Supplementary Figure 1**).

#### 3.4.2 Sino-nasal outcome test-22

A total of 8 RCTs were included (24–30, 33). Compared with the placebo group, the anti-IL-5 therapy group demonstrated significantly lower SNOT-22 scores and marked improvement (WMD: -9.57, 95% CI: -11.03 ~ -8.11, P < 0.00001, I^2^ = 80%)(**Supplementary Figure 4**). The results of meta-analysis were not prone to change significantly due to the change of the number of studies and were robust (**Supplementary Figure 5**). An evaluation of publication bias was conducted using funnel plots in relation to SNOT-22 score (**Supplementary Figure 6**) and the bilaterally symmetrical funnel plot showed no significant evidence of publication bias.

Among the results of the subgroup analysis, benralizumab (WMD: -8.85, 95% CI: -9.80 ~ -7.91, P < 0.00001, I^2^ = 0%), mepolizumab (WMD: -11.63, 95% CI: -14.04 ~ -9.21, P < 0.00001, I^2^ = 30%) and depemokimab (WMD: -8.10, 95% CI: -8.48 ~ -7.72, P < 0.00001) all demonstrated superiority over placebo in terms of SNOT-22 scores. (**Supplementary Figure 4**).

#### 3.4.3 Nasal blockage score

A total of 6 RCTs were included (24, 25, 28–30, 33). The anti-IL-5 therapy demonstrated superior efficacy in reducing NBS compared to the placebo group (SMD: -1.75; 95% CI: -3.13 ~ -0.36, P = 0.01, I^2^ = 99%) (**Supplementary Figure 7**).

In the results of the subgroup analysis, benralizumab and depemokimab reduced the NCS (benralizumab: SMD: -0.32; 95% CI: -0.53 ~ -0.12; P = 0.002, I^2^ = 0%; depemokimab SMD: -3.99; 95% CI: -4.31 ~ -3.68; P < 0.00001), but not mepolizumab (SMD: -1.82; 95% CI: -3.84 ~ -0.20; P = 0.08, I^2^ = 99%). (**Supplementary Figure 7**).

#### 3.4.4 Loss of smell score

A total of 6 RCTs were included (24, 25, 28–30, 33). A remarkable difference between anti-IL-5 therapy and placebo, with the former providing superior efficacy in reducing LSS (SMD: -1.68; 95% CI: -3.13 ~ -0.24, P = 0.02, I^2^ = 99%)(**Supplementary Figure 8**).

Among the results of the subgroup analysis, benralizumab and depemokimab reduced the LSS (benralizumab: SMD: -0.27, 95% CI: -0.47 ~ -0.06, P = 0.01, I^2^ = 0%; depemokimab: SMD: -4.39, 95% CI: -4.73 ~ -4.06, P < 0.00001), but no significant difference was exhibited in mepolizumab versus placebo (SMD: -1.56, 95% CI: -3.41 ~ -0.29, P = 0.10, I^2^ = 99%). (**Supplementary Figure 8**).

#### 3.4.5 Lund-Mackay score

A total of five RCTs (24–26, 30, 33) were included. The anti-IL-5 therapy group showed a significant difference relative to the placebo group, demonstrating that anti-IL-5 therapy significantly reduced LMS (WMD: -2.00; 95% CI: -2.65 ~ -1.34, P < 0.00001, I^2^ = 97%) (**Supplementary Figure 9**).

In the outcome of the subgroup analysis, benralizumab (WMD: -1.84, 95% CI: -3.11 ~ -0.57, P = 0.004, I^2^ = 57%), mepolizumab (WMD: -1.64, 95% CI: -1.78 ~ -1.50, P < 0.00001) and depemokimab (WMD: −2.50, 95% CI: -2.56 ~ -2.44, P < 0.00001) all contributed to a significant reduction in LMS. (**Supplementary Figure 9**).

#### 3.4.6 University of Pennsylvania smell identification test

The UPSIT was assessed only in 2 RCTs (Tversky et al. (26) and Han et al. (28)). The anti-IL-5 therapy group significantly improved the UPSIT score compared with the placebo group (WMD: 2.09, 95% CI: 0.42 ~ 3.77, P = 0.01, I^2^ = 0%). (**Supplementary Figure 10**).

#### 3.4.7 Nasal overall VAS score

The indicator was assessed only in 2 RCTs (Fujieda et al. (30) and Han et al. (28)). A remarkable difference in terms of nasal overall VAS score of anti-IL-5 therapy group compared with the placebo group (WMD: -1.54, 95% CI: -1.64 ~ -1.43, P < 0.00001, I^2^ = 0%). (**Supplementary Figure 11**).

#### 3.4.8 Nasal composite VAS score

The indicator was assessed only in 2 RCTs (Fujieda et al. (30) and Han et al. (28)). A significant difference was observed between the anti-IL-5 therapy group and the placebo group in the nasal composite VAS score (WMD: -1.28, 95% CI: -1.66 ~ -0.19, P < 0.00001, I^2^ = 0%). (**Supplementary Figure 12**).

#### 3.4.9 Number of first-time NP surgery patients

The number of first-time NP surgery patients was assessed only in 3 RCTs (24, 28, 33). The anti-IL-5 therapy group significantly reduced the time to first NP surgery compared with the placebo group (RR: 0.64, 95% CI: 0.41 ~ 1.00, P = 0.05, I^2^ = 69%). (**Supplementary Figure 13**).

#### 3.4.10 Number of systemic corticosteroids (≥1 course) patients

The number of systemic corticosteroids (≥1 course) patients was assessed only in 3 RCTs (24, 28, 33). The anti-IL-5 therapy group significantly reduced the systemic corticosteroids (≥1 course) for NP score compared with the placebo group (RR: 0.73, 95% CI: 0.62 ~ 0.86, p = 0.0002, I^2^ = 0%). (**Supplementary Figure 14**).

#### 3.4.11 Adverse events

The results of the meta-analysis of all AEs were summarized in **Table 3**. Results showed that, overall, there was no significant difference between the anti-IL-5 therapy group and the placebo group in the occurrence of any treatment-related AEs (RR: 1.01, 95% CI: 0.96 ~ 1.06, p = 0.65, I^2^ = 0%) (**Supplementary Figure 15**) and SAEs (RR: 0.86, 95% CI: 0.60 ~ 1.23, p = 0.40, I^2^ = 15%)(**Supplementary Figure 16**). For the adverse event asthma, there was a significant difference between the anti-IL-5 therapy group and the placebo group(RR: 0.48, 95% CI: 0.31 ~ 0.74, p = 0.001, I^2^ = 12%)(**Supplementary Figure 20**); no significant differences were observed for other adverse events(**Supplementary Figures 17–19**, **21–28**).

**Table 3:** Results for adverse events.

Outcomes	No. of studies	Sample size		Heterogeneity		Overall effect size	95% CI of overall effect	P value
		Experimental	Control	I^2^(%)	P value			
Any on-treatment AE (≥1 AE)	8	933	880	0	0.90	RR = 1.01	0.96 ~ 1.06	0.65
Serious Adverse Events (SAEs)	7	922	880	15	0.32	RR = 0.86	0.60 ~ 1.23	0.40
Adverse events								
Nasopharyngitis	7	689	614	0	0.80	RR = 0.92	0.73 ~ 1.16	0.49
Upper respiratory tract infection	6	476	436	23	0.27	RR = 0.76	0.46 ~ 1.25	0.28
Headache	7	689	614	5	0.39	RR = 8.82	0.62 ~ 1.08	0.16
Asthma	5	573	547	12	0.34	RR = 0.48	0.31 ~ 0.74	0.001
Epistaxis	2	259	253	0	0.38	RR = 0.84	0.46 ~ 1.53	0.56
Back pain	3	343	338	39	0.19	RR = 1.19	0.66 ~ 2.12	0.57
Oropharyngeal pain	2	259	253	0	0.94	RR = 1.54	0.80 ~ 2.93	0.19
Cough	2	259	253	0	0.83	RR = 0.55	0.25 ~ 1.22	0.14
Arthralgia	2	259	253	1	0.31	RR = 1.95	0.85 ~ 4.49	0.11
Pyrexia	4	256	200	0	0.79	RR = 1.24	0.56 ~ 2.77	0.58
Sinusitis	4	334	280	46	0.13	RR = 0.73	0.41 ~ 1.29	0.28
Influenza	3	172	115	52	0.12	RR = 1.26	0.16 ~ 9.94	0.83

## 4 Discussion

### 4.1 General interpretation of the results

This meta-analysis of 10 RCTs (n=1,859) confirms that anti-IL-5 mAbs are effective as second-line therapy for CRSwNP, significantly improving CRSwNP key outcomes.

In terms of effectiveness, the NPS, SNOT-22 score, NBS, LSS, LMS, UPSIT, nasal overall VAS score, and nasal composite VAS score were used to compare the two treatments. Contemporary guidelines have reached a consensus on the evaluation value of NPS and LMS (34). Psychometric analyses supported the validity, reliability, and responsiveness of the five domains of SNOT-22 (nasal, ear/face, sleep, function, and mood) used to assess symptoms and impact on HRQoL in patients with CRSwNP (35). The UPSIT is a 40-item odor test that is widely used to assess hyposmia (36). The results of this meta-analysis clearly showed that compared with placebo, the anti-IL-5 mab treatment regimens of the included RCTs can significantly improve the scores of these assessments. Especially the magnitude of improvement in NPS (WMD –0.58) and SNOT-22 (–9.57) exceeds the minimal clinically important difference (MCID) for these instruments (NPS MCID ~0.5, SNOT-22 MCID ~8.9), supporting clinical relevance (35, 37). However, all measures showed significant heterogeneity except for the sample size heterogeneity for UPSIT and nasal overall VAS scores, which were not significant. Heterogeneity likely arises from multiple sources: (1) Drug molecule: benralizumab depletes eosinophils via antibody-dependent cell-mediated cytotoxicity, whereas mepolizumab and reslizumab neutralize IL-5, and depemokimab has ultra-long duration (32, 33, 38, 39); these mechanistic differences may translate into varying efficacy profiles. (2) Dosage and treatment duration: trials used different regimens (e.g., mepolizumab 100 mg SC q4w vs 750 mg IV q4w; benralizumab 30 mg SC q4w or q8w). Treatment duration ranged from 8 to 52 weeks, and longer treatment may yield greater improvements. (3) Baseline disease severity: trials enrolled patients with moderate-to-severe CRSwNP, but entry criteria varied (e.g., prior surgery rates, baseline NPS thresholds). (4) Outcome scale heterogeneity: for NBS and LSS, some trials used a 0–3 VRS and others a 0–10 VAS, requiring SMD, which can inflate heterogeneity. (5) Population differences: early trials included predominantly Caucasian patients with type 2 inflammation; recent trials (e.g., MERIT (30)) enrolled Asian populations, where mixed inflammatory phenotypes exist. The results showed that the anti-IL-5 mab treatment for CRSwNP was more effective than placebo. The mechanism was the anti-IL-5 antibody prevents the cytokine IL-5 from binding to the α chain of the IL-5 receptor complex on the surface of eosinophils, thereby reducing the level and activation state of eosinophils (40).

For safety, the complications identified in the included RCTs mainly included nasopharyngitis, upper respiratory tract infection, headache, and asthma. The results of 7 RCTs (24, 25, 27–30, 32) indicated that there was no significant difference in the occurrence of any on-treatment AEs between the anti-IL-5 mab group and the control group after treatment, and the incidence rates were similar. Furthermore, the results of 7 RCTs (24, 27–31, 33) indicated that there was also no significant difference in the incidence of SAEs during treatment between the anti-IL-5 mab group and the control group. The results of the 10 RCTs (24–33) included confirmed that the incidence of AEs in the two groups (anti-IL-5 mab VS placebo) was similar, and anti-IL-5 mab was safe and well tolerated. For nasopharyngitis, supportive care and nasal irrigation can be carried out. Usually, no adjustment of anti-IL-5 treatment is necessary. For upper respiratory tract infections, supportive care and antibiotic treatment when necessary can be provided. In cases of severe bacterial infection, anti-IL-5 treatment can be temporarily postponed. For adverse reaction of headache, it may be related to multiple factors and the administration method. The causes can be evaluated and symptomatic pain relief can be provided. Rarely, adjustment of anti-IL-5 treatment is needed. It is only used for severe intolerance. For asthma, the activity of underlying diseases is the main cause. Treatment according to the GINA guidelines (41) can be carried out. Anti-IL-5 treatment adjustment should not be stopped. Instead, it should continue to control long-term risks. To sum up, anti-IL-5 mab is effective and safe for the treatment of CRSwNP, greatly reducing concerns about long-term IL-5 inhibition. It should be noted that among the AEs in CRSwNP patients treated with anti-IL-5 mab, only asthma showed a significant difference compared with the control group, with fewer events occurring. The reduction in asthma events (RR 0.48) in the active arm is notable. Anti-IL-5 agents are established treatments for severe eosinophilic asthma, so this finding likely reflects concomitant asthma improvement. However, it may also indicate selection bias: trials often included patients with comorbid asthma to enrich for eosinophilic inflammation. This is consistent with the result of Bachert, Claus et al (24), which showed that CRSwNP with comorbid asthma had better responses. Moreover, Gevaert, Philippe et al (31) showed that IL-5 inhibition is a potential treatment for severe eosinophilic nasal polyps.

Subgroup analyses by drug revealed that benralizumab and depemokimab consistently improved all outcomes, while mepolizumab showed significant effects for most but with high heterogeneity, and reslizumab did not reach significance for NPS. The reslizumab finding derives from a single phase I trial with only 8 patients per arm (32). The lack of statistical significance is almost certainly a type II error (insufficient power), not evidence of inefficacy. Depemokimab, while showing large effects, is supported by only one phase III program (ANCHOR-1 and -2) (33); replication in diverse populations is warranted. Leave-one-out sensitivity analysis of the NPS (**Supplementary Figure 2**) revealed that the overall estimate remained stable, but heterogeneity decreased when the depemokimab trial (31) was removed (I² dropped from 83% to 48%), suggesting that the ultra-long-acting drug or its large sample size contributed to heterogeneity. By targeting the alpha subunit of IL-5Rα, benalizumab blocks IL-5 signaling and targets these cells to enhance antibody-dependent cytotoxicity, resulting in rapid and nearly complete clearance of blood eosinophils and a reduction in basophil counts (24). Subcutaneous injection of mepolizumab is the only approved anti-IL-5 therapy to date in CRSwNP (39). In patients with CRSwNP and asthma, dupilumab improves asthma symptoms and reduces the need for SCS which might help patients avoid SCS-related side effects (42). The RCT results of Han, Joseph K et al (28), Bachert C et al (29), Fujieda, S et al (30), Tversky, Jody et al (26) and Gevaert, Philippe et al (33) showed that mepolizumab or benralizumab can reduce the risk of nasal polyp surgery or the use of systemic corticosteroids. Additionally, compared to the 2022 review (43), this meta-analysis includes a previously unreported RCT on the drug depemokimab. Gevaert, Philippe et al. (33) confirmed that depemokimab—the world’s first ultra-long-acting anti-IL-5 biologic administered twice yearly—is effective and well-tolerated for chronic rhinosinusitis with nasal polyps (CRSwNP), significantly reducing the treatment burden.

### 4.2 Limitations of the evidence included in the review

The included trials had several limitations that affected the generalizability of the findings. First, among the 10 RCTs, Phase I and II trials had smaller sample sizes, limiting the statistical power of subgroup analyses. Phase III trials had larger samples but were restricted to specific populations (e.g., >60% with prior polypectomy), excluding treatment-naive patients. Second, all trials evaluated efficacy only within 52 weeks, despite CRSwNP being a chronic, recurrent disease. Long-term outcome data (e.g., polyp recurrence rates) spanning 2–5 years were lacking, which is critical for assessing long-term safety. Third, early trials primarily enrolled Caucasian participants (80-90% with Type II inflammation), with Asian population data emerging only recently. This limits extrapolation to non-Caucasian cohorts, where mixed pathological phenotypes (Type II + neutrophilic) are more prevalent. Fourth, total NPS was uniformly used, but symptom severity was assessed via different tools: verbal response scale (VRS) in ANCHOR (33) and visual analog scale (VAS) in MERIT (30), complicating direct meta-analytic pooling.

### 4.3 Limitations of the review processes used

First, potential publication bias cannot be ruled out: only published randomized controlled trials were included, and negative or neutral results (e.g., small phasey trials and II trials of anti-IL-5 mAbs) may be underreported, overestimating efficacy. Funnel plot asymmetry would be needed to quantify this, but were not feasible due to limited trial numbers for some endpoints (e.g., depemokimab has only 1 trials). Second, the literature search strategy might be flawed, potentially leading to literature omission. Third, though we had tried to contact corresponding authors, many missing critical data remained unavailable. Fourth, a full assessment of the effects of confounding factors was hampered by raw data limitations.

### 4.4 Implications for future research and clinical practice

The limitations identified in both the included evidence and our review processes have significant ramifications, pointing toward clear directions for future research: (1) Prioritize Large, Long-Term, and Pragmatic Trials. (2) A need for trials that proactively enroll diverse ethnic populations. (3) Standardization of Outcome Measures, which will facilitate robust meta-analyses and direct cross-trial comparisons in the future, reducing heterogeneity and increasing the strength of evidence. (4) Promote Data Transparency and Collaboration, which would mitigate the problems caused by aggregated data reporting and missing information.

Our findings have critical clinical relevance for managing severe, refractory CRS. Anti-IL-5 mAbs should be considered as second-line therapy for patients with eCRS (≥15 eosinophils/hpf) who fail maximal medical therapy (intranasal corticosteroids + short-course oral corticosteroids) or are intolerant to systemic steroids. For CRSwNP patients, anti-IL-5 may also reduce the need for repeated endoscopic sinus surgery (ESS).

Future research should focus on three areas: (1) long-term efficacy and recurrence prevention: Most trials follow patients for 52 weeks; studies exceeding 1 year are needed to assess polyp recurrence. (2) combination therapy: for the residual symptoms of non-responders, combination therapy with other drugs may be considered, which may be effective in regulating mixed type 2/neutrophilic inflammation, especially for Asian patients. (3) real-world validation: RCTs often exclude complex patients (e.g. comorbidities); real-world studies should confirm efficacy in diverse populations and refine biomarker thresholds. In short, Future research should address long-term outcomes, combination therapy and real-world applicability, while reduced publication bias are needed to strengthen conclusions.

## 5 Conclusion

This meta-analysis demonstrates that anti-IL-5 mAbs are safe and efficacious for CRSwNP treatment, yielding notable improvements in symptoms, polyp size, and quality of life. The first-line preferred therapy for CRSwNP remains “nasal corticosteroids in combination with nasal saline irrigation”. Subsequent therapeutic strategies require individualization and escalation according to patients’ disease classification, severity, and response to initial treatment. Anti-IL-5 mAbs may also serve as a second-line therapeutic option. Despite existing limitations (including heterogeneity and insufficient long-term data), the results endorse incorporating anti-IL-5 mAbs into personalized CRSwNP management protocols. This calls for future research to broaden their utility and optimize patient selection criteria.

## Data Availability

The original contributions presented in the study are included in the article/**Supplementary Material**. Further inquiries can be directed to the corresponding author.

## References

[1] Singh AK Kanaujia SK Khan MS . Determination of sensitivity and specificity of diagnostic nasal endoscopy compared to computed tomography scan of nasal and paranasal sinuses among patients suffering from chronic rhinosinusitis. Cureus. (2025) 17:e87760. doi: 10.7759/cureus.87760. PMID: 40792313 PMC12336917

[2] Fokkens WJ Lund VJ Hopkins C Hellings PW Kern R Reitsma S . European position paper on rhinosinusitis and nasal polyps 2020. Rhinology. (2020) 58:1–464. doi: 10.4193/rhin20.600. PMID: 32077450

[3] Hastan D Fokkens WJ Bachert C Newson RB Bislimovska J Bockelbrink A . Chronic rhinosinusitis in Europe--an underestimated disease. A GA²LEN study. Allergy. (2011) 66:1216–23. doi: 10.1111/j.1398-9995.2011.02646.x. PMID: 21605125

[4] Beule A . Epidemiology of chronic rhinosinusitis, selected risk factors, comorbidities, and economic burden. GMS Curr Topics Otorhinolaryngol Head Neck Surg. (2015) 14:Doc11. doi: 10.3205/cto000126, PMID: 26770285 PMC4702060

[5] Alobid I Benítez P Bernal-Sprekelsen M Roca J Alonso J Picado C . Nasal polyposis and its impact on quality of life: comparison between the effects of medical and surgical treatments. Allergy. (2005) 60:452–8. doi: 10.1111/j.1398-9995.2005.00725.x. PMID: 15727575

[6] Sahlstrand-Johnson P Ohlsson B Von Buchwald C Jannert M Ahlner-Elmqvist M . A multi-centre study on quality of life and absenteeism in patients with CRS referred for endoscopic surgery. Rhinology. (2011) 49:420–8. doi: 10.4193/rhino11.101. PMID: 21991567

[7] Bhattacharyya N . Contemporary assessment of the disease burden of sinusitis. Allergy Rhinol (Providence RI). (2010) 1:8. doi: 10.2500/ajra.2009.23.3355. PMID: 28569228

[8] Peters AT Spector S Hsu J Hamilos DL Baroody FM Chandra RK . Diagnosis and management of rhinosinusitis: a practice parameter update. Ann Allergy Asthma Immunol: Off Publ Am Coll Allergy Asthma Immunol. (2014) 113:347–85. doi: 10.1016/j.anai.2014.07.025. PMID: 25256029

[9] Lund VJ Flood J Sykes AP Richards DH . Effect of fluticasone in severe polyposis. Arch Otolaryngology--Head Neck Surg. (1998) 124:513–8. doi: 10.1001/archotol.124.5.513. PMID: 9604976

[10] Poetker DM Jakubowski LA Lal D Hwang PH Wright ED Smith TL . Oral corticosteroids in the management of adult chronic rhinosinusitis with and without nasal polyps: an evidence-based review with recommendations. Int Forum Allergy Rhinol. (2013) 3:104–20. doi: 10.1007/978-3-319-16724-4_15. PMID: 22887970

[11] Fokkens WJ De Corso E Backer V Bernal-Sprekelsen M Bjermer L von Buchwald C . EPOS2020/EUFOREA expert opinion on defining disease states and therapeutic goals in CRSwNP. Rhinology. (2024) 62:287–98. doi: 10.4193/rhin23.415. PMID: 38217529

[12] Fokkens WJ Lund VJ Hopkins C Hellings PW Kern R Reitsma S . Executive summary of EPOS 2020 including integrated care pathways. Rhinology. (2020) 58:82–111. doi: 10.4193/rhin20.601. PMID: 32226949

[13] Foerster-Ruhrmann U Jurkov M Szczepek AJ Bergmann KC Fluhr JW Olze H . Biologics reduce symptoms of alcohol intolerance better than aspirin desensitization in patients with N-ERD and nasal polyps. Biomedicines. (2024) 12:1025–37. doi: 10.3390/biomedicines12051025. PMID: 38790987 PMC11118606

[14] Han SA Yoon J Kim BH Park JH Lim YS Kim YB . Real-world treatment of chronic rhinosinusitis with dupilumab and omalizumab: results from the Korean National Health Insurance Service database. Rhinology. (2025) 63:172–9. doi: 10.4193/rhin24.209. PMID: 39968898

[15] Loperfido A Cavaliere C Ciofalo A Bugani M Begvarfaj E Millarelli S . Remission in chronic rhinosinusitis with nasal polyps treated with biologics: a real-life experience. Eur Arch Oto-Rhino-Laryngol: Off J Eur Fed Oto-Rhino-Laryngol Societies (EUFOS): Affiliated German Soc For Oto-Rhino-Laryngol - Head Neck Surg. (2025) 282:4681–90. doi: 10.1007/s00405-025-09619-y. PMID: 40759784

[16] Priessnitz J Jung J Han JK Lam KK . Evaluating the efficacy and safety of tezepelumab in the treatment of chronic rhinosinusitis with nasal polyps. Immunotherapy. (2025) 17(13):903–12. doi: 10.1080/1750743x.2025.2567844. PMID: 41084787 PMC12691544

[17] Bachert C Zhang N Cavaliere C Weiping W Gevaert E Krysko O . Biologics for chronic rhinosinusitis with nasal polyps. J Allergy Clin Immunol. (2020) 145:725–39. doi: 10.1016/j.jaci.2020.01.020. PMID: 32145872

[18] Chikumoto A Oishi K Hamada K Hirano T Kakugawa T Kanesada K . Sequential biotherapy targeting IL-5 and IL-4/13 in patients with eosinophilic asthma with sinusitis and otitis media. Biomolecules. (2022) 12 522–8. doi: 10.3390/biom12040522. PMID: 35454111 PMC9025540

[19] Sanderson CJ . Eosinophil differentiation factor (interleukin-5). Immunol Ser. (1990) 49:231–56. 2090253

[20] Murata Y Takaki S Migita M Kikuchi Y Tominaga A Takatsu K . Molecular cloning and expression of the human interleukin 5 receptor. J Exp Med. (1992) 175:341–51. doi: 10.1084/jem.175.2.341. PMID: 1732409 PMC2119102

[21] Sanderson CJ . Interleukin-5, eosinophils, and disease. Blood. (1992) 79:3101–9. doi: 10.1182/blood.v79.12.3101.bloodjournal79123101. PMID: 1596561

[22] Liu LY Sedgwick JB Bates ME Vrtis RF Gern JE Kita H . Decreased expression of membrane IL-5 receptor alpha on human eosinophils: I. Loss of membrane IL-5 receptor alpha on airway eosinophils and increased soluble IL-5 receptor alpha in the airway after allergen challenge. J Immunol (Baltimore Md: 1950). (2002) 169:6452–8. doi: 10.4049/jimmunol.169.11.6452. PMID: 12444154

[23] McBrien CN Menzies-Gow A . The biology of eosinophils and their role in asthma. Front Med (Lausanne). (2017) 4:93. doi: 10.3389/fmed.2017.00093. PMID: 28713812 PMC5491677

[24] Bachert C Han JK Desrosiers MY Gevaert P Heffler E Hopkins C . Efficacy and safety of benralizumab in chronic rhinosinusitis with nasal polyps: a randomized, placebo-controlled trial. J Allergy Clin Immunol. (2022) 149:1309–1317.e12. doi: 10.1016/j.jaci.2021.08.030. PMID: 34599979

[25] Takabayashi T Asaka D Okamoto Y Himi T Haruna S Yoshida N . A phase II, multicenter, randomized, placebo-controlled study of benralizumab, a humanized anti-IL-5R alpha monoclonal antibody, in patients with eosinophilic chronic rhinosinusitis. Am J Rhinol Allergy. (2021) 35:861–70. doi: 10.1177/19458924211009429. PMID: 33840229

[26] Tversky J Lane AP Azar A . Benralizumab effect on severe chronic rhinosinusitis with nasal polyps (CRSwNP): a randomized double-blind placebo-controlled trial. Clin Exp Allergy: J Br Soc For Allergy Clin Immunol. (2021) 51:836–44. doi: 10.1111/cea.13852. PMID: 33595845

[27] Canonica GW Harrison TW Chanez P Menzella F Louis R Cosio BG . Benralizumab improves symptoms of patients with severe, eosinophilic asthma with a diagnosis of nasal polyposis. Allergy. (2022) 77:150–61. doi: 10.1111/all.14902. PMID: 33978983

[28] Han JK Bachert C Fokkens W Desrosiers M Wagenmann M Lee SE . Mepolizumab for chronic rhinosinusitis with nasal polyps (SYNAPSE): a randomised, double-blind, placebo-controlled, phase 3 trial. Lancet Respir Med. (2021) 9:1141–53. doi: 10.1016/s2213-2600(21)00097-7. PMID: 33872587

[29] Bachert C Sousa AR Lund VJ Scadding GK Gevaert P Nasser S . Reduced need for surgery in severe nasal polyposis with mepolizumab: randomized trial. J Allergy Clin Immunol. (2017) 140:1024–1031.e14. doi: 10.1016/j.jaci.2017.05.044. PMID: 28687232

[30] Fujieda S Wang C Yoshikawa M Asako M Suzaki I Bachert C . Mepolizumab in CRSwNP/ECRS and NP: the phase III randomised MERIT trial in Japan, China, and Russia. Rhinology. (2024) 62:576–89. doi: 10.4193/rhin24.156. PMID: 39058315

[31] Gevaert P Van Bruaene N Cattaert T Van Steen K Van Zele T Acke F . Mepolizumab, a humanized anti-IL-5 mAb, as a treatment option for severe nasal polyposis. J Allergy Clin Immunol. (2011) 128:989–95.e1-8. doi: 10.1016/j.jaci.2011.07.056. PMID: 21958585

[32] Gevaert P Lang-Loidolt D Lackner A Stammberger H Staudinger H Van Zele T . Nasal IL-5 levels determine the response to anti-IL-5 treatment in patients with nasal polyps. J Allergy Clin Immunol. (2006) 118:1133–41. doi: 10.1016/j.jaci.2006.05.031. PMID: 17088140

[33] Gevaert P Desrosiers M Cornet M Mullol J De Corso E Keles Turel N . Efficacy and safety of twice per year depemokimab in chronic rhinosinusitis with nasal polyps (ANCHOR-1 and ANCHOR-2): phase 3, randomised, double-blind, parallel trials. Lancet (London England). (2025) 405:911–26. doi: 10.1016/s0140-6736(25)00197-7. PMID: 40037388

[34] Kaper NM van der Heijden G Cuijpers SH Stokroos RJ Aarts MCJ . A comparison of international clinical practice guidelines on adult chronic rhinosinusitis shows considerable variability of recommendations for diagnosis and treatment. Eur Arch Oto-Rhino-Laryngol: Off J Eur Fed Oto-Rhino-Laryngol Societies (EUFOS): Affiliated German Soc For Oto-Rhino-Laryngol - Head Neck Surg. (2020) 277:659–68. doi: 10.1007/s00405-019-05752-7. PMID: 31845037

[35] Khan AH Reaney M Guillemin I Nelson L Qin S Kamat S . Development of sinonasal outcome test (SNOT-22) domains in chronic rhinosinusitis with nasal polyps. Laryngoscope. (2022) 132:933–41. doi: 10.1002/lary.29766. PMID: 34437720 PMC9292332

[36] Joseph T Auger SD Peress L Rack D Cuzick J Giovannoni G . Screening performance of abbreviated versions of the UPSIT smell test. J Neurol. (2019) 266:1897–906. doi: 10.1007/s00415-019-09340-x. PMID: 31053960 PMC6647236

[37] Liu CM Fischer JL Overdevest JB Zemke AC Strand MJ Gudis DA . Psychometric validity of the 22-item sinonasal outcome test in cystic fibrosis. J Cyst Fibros. (2026) 14:S1569-1993(25)02553-6. doi: 10.1016/j.jcf.2025.12.022. PMID: 41535163

[38] Kolbeck R Kozhich A Koike M Peng L Andersson CK Damschroder MM . MEDI-563, a humanized anti-IL-5 receptor alpha mAb with enhanced antibody-dependent cell-mediated cytotoxicity function. J Allergy Clin Immunol. (2010) 125:1344–1353.e2. doi: 10.1016/j.jaci.2010.04.004. PMID: 20513525

[39] Lombardi C Comberiati P Ridolo E Cottini M Yacoub MR Casagrande S . Anti-IL-5 pathway agents in eosinophilic-associated disorders across the lifespan. Drugs. (2024) 84:661–84. doi: 10.1007/s40265-024-02037-0. PMID: 38849701 PMC11196311

[40] Rothenberg ME . Humanized anti-IL-5 antibody therapy. Cell. (2016) 165:509. doi: 10.1016/j.cell.2016.04.020. PMID: 27104969

[41] Mauer Y Taliercio RM . Managing adult asthma: the 2019 GINA guidelines. Cleve Clin J Med. (2020) 87:569–75. doi: 10.3949/ccjm.87a.19136. PMID: 32868307

[42] Gurnell M Radwan A Bachert C Lugogo N Cho SH Nash S . Dupilumab reduces asthma disease burden and recurrent SCS use in patients with CRSwNP and coexisting asthma. J Asthma Allergy. (2024) 17:1–8. doi: 10.1016/j.jaci.2021.12.427. PMID: 38250137 PMC10799571

[43] Wang Q Sun Q Chen Q Li H Liu D Wu Q . Efficacy and safety of anti-interleukin-5 therapies in chronic rhinosinusitis with nasal polyps: a systematic review and meta-analysis of randomized controlled trials. Int Arch Allergy Immunol. (2022) 183:732–43. doi: 10.1159/000521859. PMID: 35108711

