# Supplementary material for: Safety and efficacy of anti-IL-5 monoclonal antibodies as second-line therapy for chronic rhinosinusitis with nasal polyps: a meta-analysis
Source: Front Immunol. 2026 Mar 30;17:1746573. doi: 10.3389/fimmu.2026.1746573 (PMC13070943; doi:10.3389/fimmu.2026.1746573)

Figure S1. Forest plot of the meta-analysis for NPS

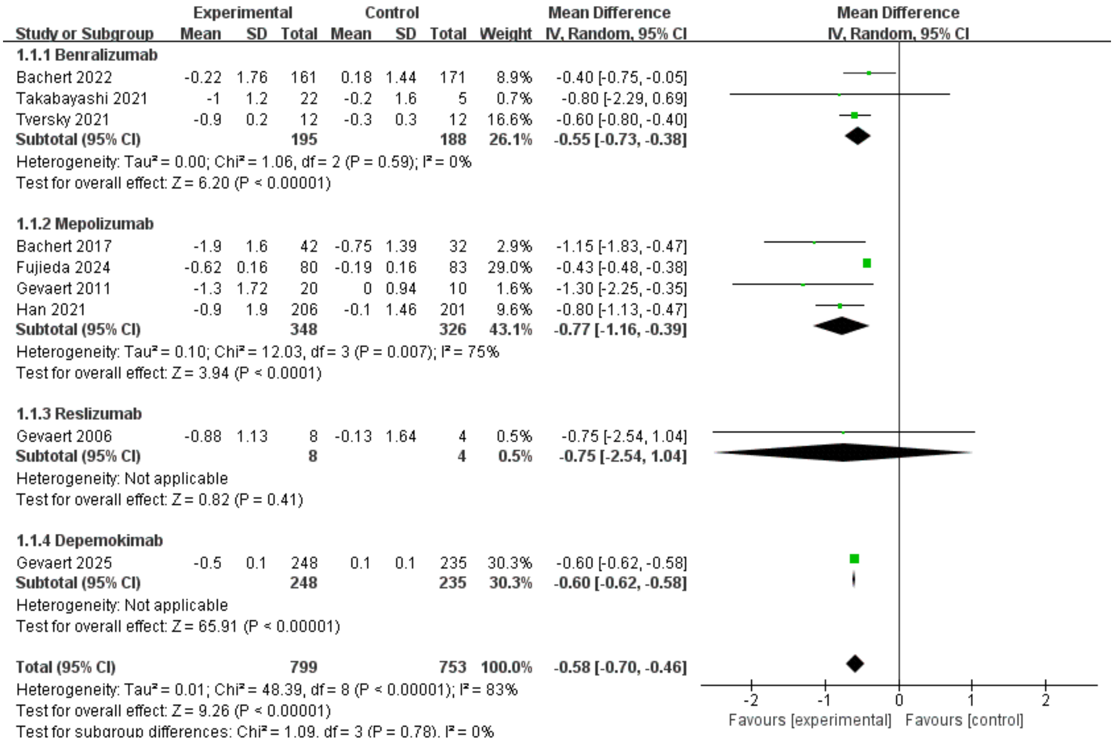

Figure S2. Sensitivity Analysis of NPS

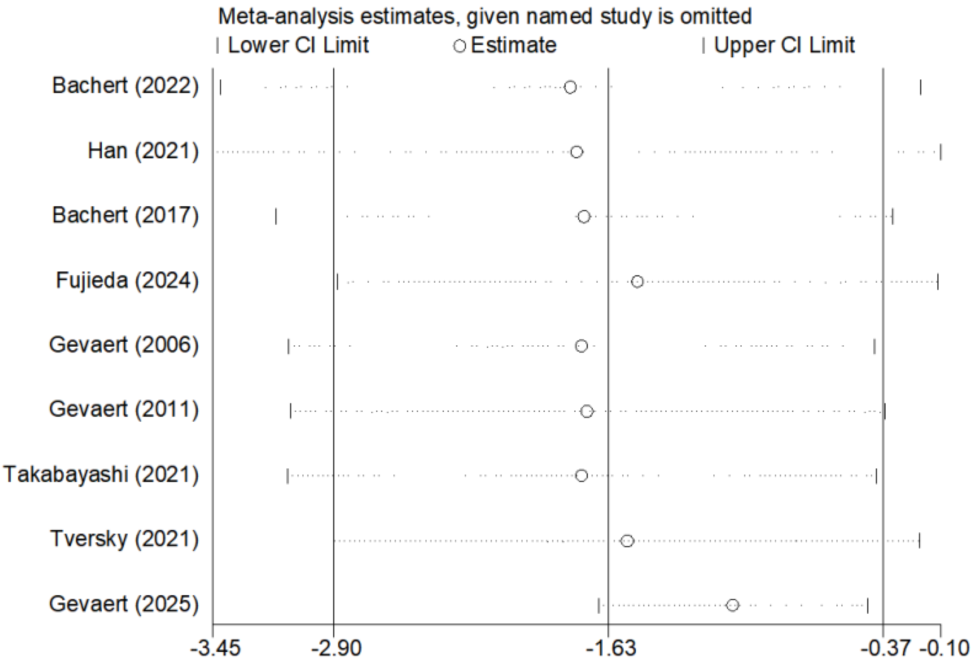

Figure S3. funnel plot for NPS

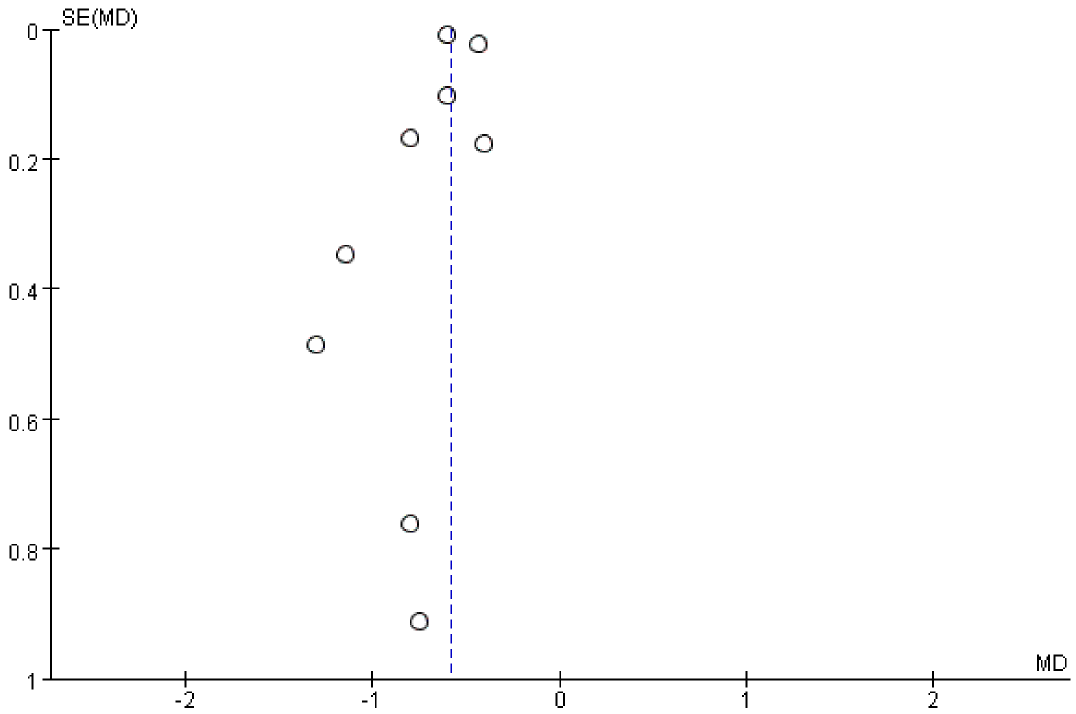

Figure S4. Forest plot of the meta-analysis for SNOT-22 score

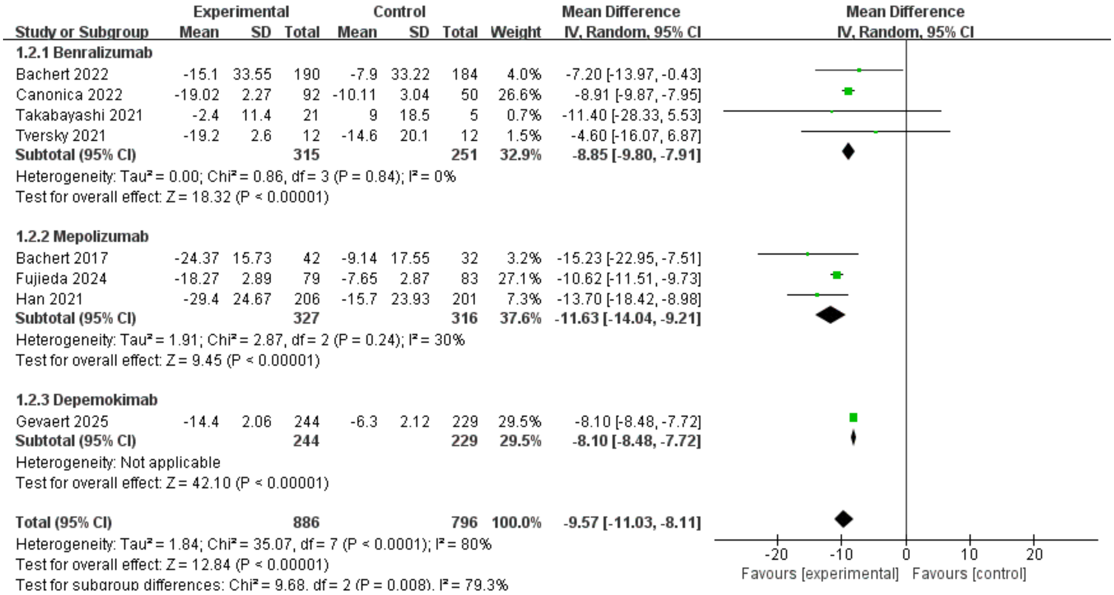

Figure S5. Sensitivity Analysis of SNOT-22 score

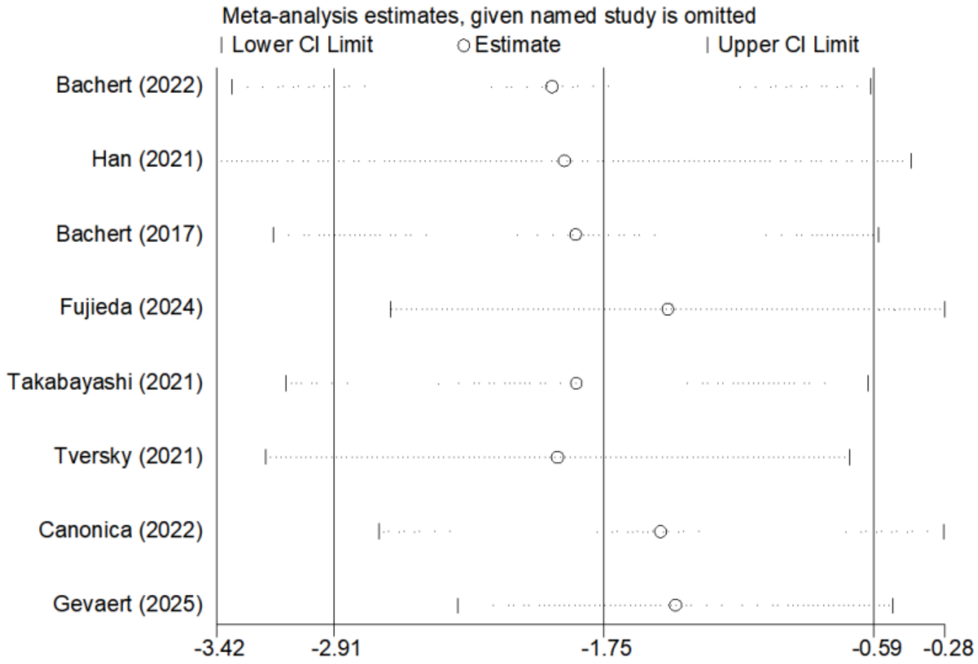

Figure S6. funnel plot for SNOT-22 score

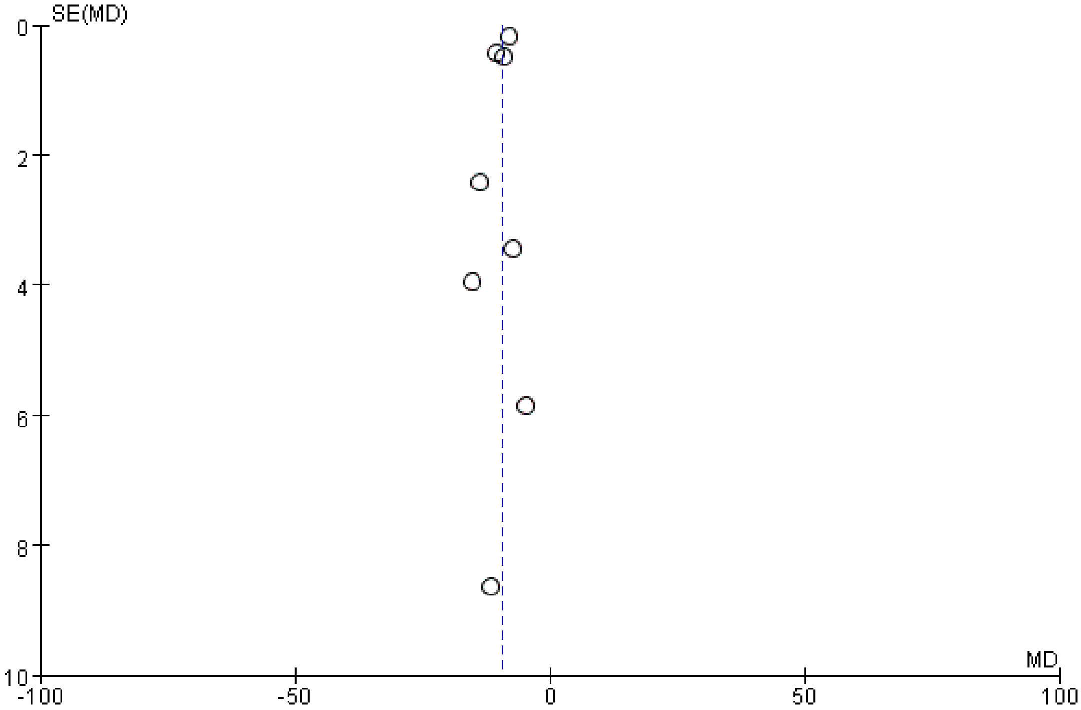

Figure S7. Forest plot of the meta-analysis for NBS

Study or Subgroup	Experimental Mean	Experimental SD	Experimental Total	Control Mean	Control SD	Control Total	Weight	Std. Mean Difference IV, Random, 95% CI
**1.3.1 Benralizumab**								
Bachert 2022	-0.68	1.03	179	-0.38	0.91	175	17.0%	-0.31 [-0.52, -0.10]
Takabayashi 2021	0.01	2.64	21	1.81	3.14	5	15.7%	-0.64 [-1.63, 0.35]
**Subtotal (95% CI)**			**200**			**180**	**32.7%**	**-0.32 [-0.53, -0.12]**
Heterogeneity: Tau² = 0.00; Chi² = 0.41, df = 1 (P = 0.52); I² = 0%								
Test for overall effect: Z = 3.08 (P = 0.002)								
**1.3.2 Mepolizumab**								
Bachert 2017	-2.63	3.03	42	-0.38	3.03	31	16.7%	-0.73 [-1.21, -0.25]
Fujieda 2024	-3.23	0.34	80	-1.8	0.33	83	16.6%	-4.25 [-4.81, -3.69]
Han 2021	-4.2	3.42	206	-2.5	3.15	201	17.0%	-0.52 [-0.71, -0.32]
**Subtotal (95% CI)**			**328**			**315**	**50.4%**	**-1.82 [-3.84, 0.20]**
Heterogeneity: Tau² = 3.15; Chi² = 152.95, df = 2 (P < 0.00001); I² = 99%								
Test for overall effect: Z = 1.76 (P = 0.08)								
**1.3.3 Depemokimab**								
Gevaert 2025	-0.77	0.06	244	-0.53	0.06	227	16.9%	-3.99 [-4.31, -3.68]
**Subtotal (95% CI)**			**244**			**227**	**16.9%**	**-3.99 [-4.31, -3.68]**
Heterogeneity: Not applicable								
Test for overall effect: Z = 24.97 (P < 0.00001)								
**Total (95% CI)**			**772**			**722**	**100.0%**	**-1.75 [-3.13, -0.36]**
Heterogeneity: Tau² = 2.92; Chi² = 539.71, df = 5 (P < 0.00001); I² = 99%								
Test for overall effect: Z = 2.47 (P = 0.01)								
Test for subgroup differences: Chi² = 369.23, df = 2 (P < 0.00001), I² = 99.5%								

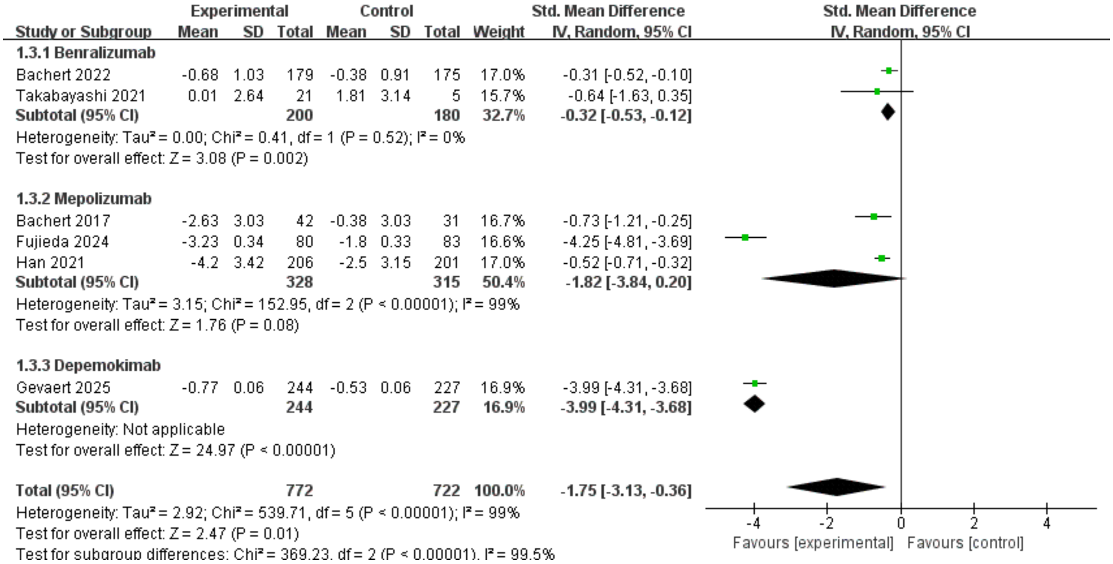

Figure S8. Forest plot of the meta-analysis for LSS

Study or Subgroup	Experimental Mean	Experimental SD	Experimental Total	Control Mean	Control SD	Control Total	Weight	Std. Mean Difference IV, Random, 95% CI
**1.4.1 Benralizumab**								
Bachert 2022	-0.39	0.79	179	-0.21	0.65	175	17.0%	-0.25 [-0.46, -0.04]
Takabayashi 2021	-1.3	2.55	21	0.68	3.07	5	15.8%	-0.73 [-1.72, 0.27]
**Subtotal (95% CI)**			**200**			**180**	**32.7%**	**-0.27 [-0.47, -0.06]**
Heterogeneity: Tau² = 0.00; Chi² = 0.84, df = 1 (P = 0.36); I² = 0%								
Test for overall effect: Z = 2.57 (P = 0.01)								
**1.4.2 Mepolizumab**								
Bachert 2017	-3.04	3.52	42	-1.2	2.93	31	16.7%	-0.55 [-1.03, -0.08]
Fujieda 2024	-1.71	0.22	80	-0.89	0.22	83	16.7%	-3.71 [-4.22, -3.20]
Han 2021	-2.8	3.61	206	-1.4	2.65	201	17.0%	-0.44 [-0.64, -0.24]
**Subtotal (95% CI)**			**328**			**315**	**50.4%**	**-1.56 [-3.41, 0.29]**
Heterogeneity: Tau² = 2.62; Chi² = 138.67, df = 2 (P < 0.00001); I² = 99%								
Test for overall effect: Z = 1.65 (P = 0.10)								
**1.4.3 Depemokimab**								
Gevaert 2025	-0.52	0.05	244	-0.3	0.05	227	16.9%	-4.39 [-4.73, -4.06]
**Subtotal (95% CI)**			**244**			**227**	**16.9%**	**-4.39 [-4.73, -4.06]**
Heterogeneity: Not applicable								
Test for overall effect: Z = 25.72 (P < 0.00001)								
**Total (95% CI)**			**772**			**722**	**100.0%**	**-1.68 [-3.13, -0.24]**
Heterogeneity: Tau² = 3.18; Chi² = 584.85, df = 5 (P < 0.00001); I² = 99%								
Test for overall effect: Z = 2.29 (P = 0.02)								
Test for subgroup differences: Chi² = 424.62, df = 2 (P < 0.00001), I² = 99.5%								

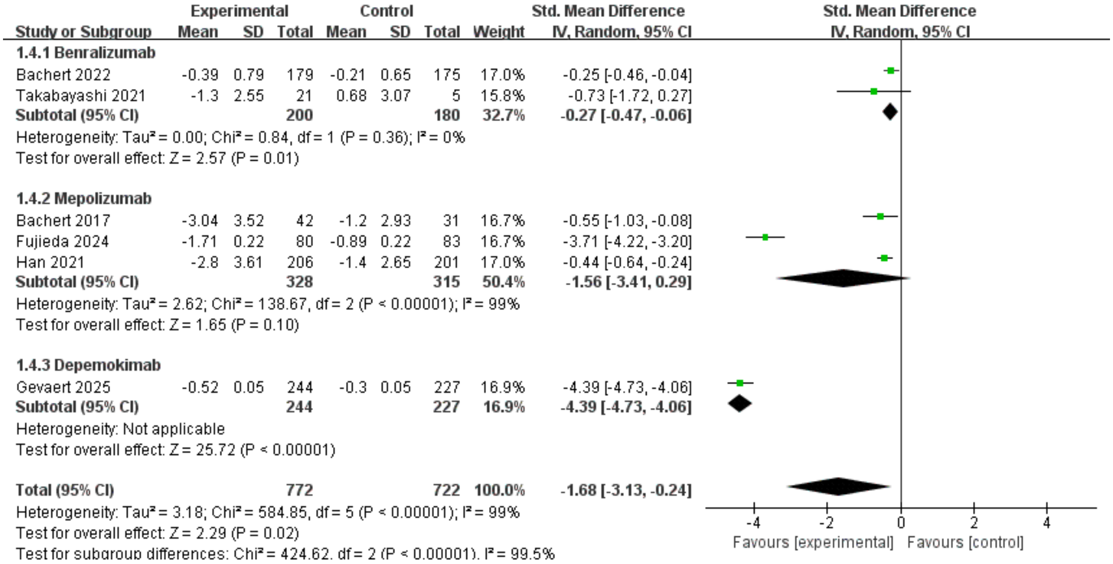

Figure S9. Forest plot of the meta-analysis for LMS

Study or Subgroup	Experimental Mean	Experimental SD	Experimental Total	Control Mean	Control SD	Control Total	Weight	Mean Difference IV, Random, 95% CI
**1.5.1 Benralizumab**								
Bachert 2022	-0.93	5.06	81	-0.2	4.2	84	12.6%	-0.73 [-2.15, 0.69]
Takabayashi 2021	-1.3	2.2	22	0.7	2.3	5	6.8%	-2.00 [-4.22, 0.22]
Tversky 2021	-4.2	1.1	12	-1.6	1.2	12	19.2%	-2.60 [-3.52, -1.68]
**Subtotal (95% CI)**			**115**			**101**	**38.6%**	**-1.84 [-3.11, -0.57]**
Heterogeneity: Tau² = 0.71; Chi² = 4.68, df = 2 (P = 0.10); I² = 57%								
Test for overall effect: Z = 2.84 (P = 0.004)								
**1.5.2 Mepolizumab**								
Fujieda 2024	-3.52	0.45	80	-1.88	0.45	83	30.5%	-1.64 [-1.78, -1.50]
**Subtotal (95% CI)**			**80**			**83**	**30.5%**	**-1.64 [-1.78, -1.50]**
Heterogeneity: Not applicable								
Test for overall effect: Z = 23.26 (P < 0.00001)								
**1.5.3 Depemokimab**								
Gevaert 2025	-3.1	0.31	248	-0.6	0.32	230	30.9%	-2.50 [-2.56, -2.44]
**Subtotal (95% CI)**			**248**			**230**	**30.9%**	**-2.50 [-2.56, -2.44]**
Heterogeneity: Not applicable								
Test for overall effect: Z = 86.63 (P < 0.00001)								
**Total (95% CI)**			**443**			**414**	**100.0%**	**-2.00 [-2.65, -1.34]**
Heterogeneity: Tau² = 0.36; Chi² = 132.92, df = 4 (P < 0.00001); I² = 97%								
Test for overall effect: Z = 5.98 (P < 0.00001)								
Test for subgroup differences: Chi² = 128.12, df = 2 (P < 0.00001), I² = 98.4%								

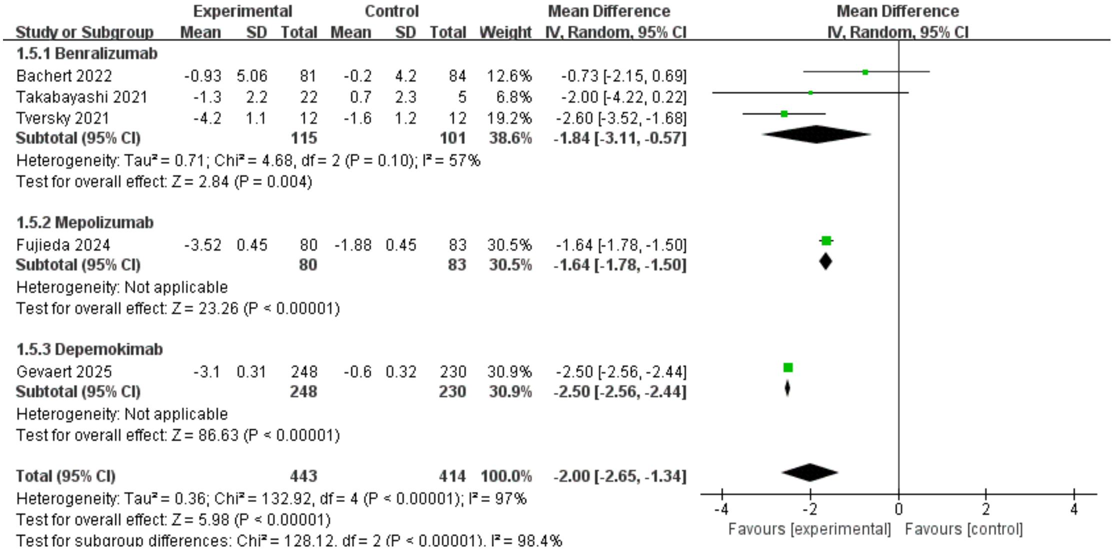

Figure S10. Forest plot of the meta-analysis for UPSIT score

Study or Subgroup	Experimental Mean	Experimental SD	Experimental Total	Control Mean	Control SD	Control Total	Weight	Mean Difference IV, Fixed, 95% CI
**1.6.1 Benralizumab**								
Tversky 2021	6.6	2.2	12	4.3	2.5	12	79.2%	2.30 [0.42, 4.18]
**Subtotal (95% CI)**			**12**			**12**	**79.2%**	**2.30 [0.42, 4.18]**
Heterogeneity: Not applicable								
Test for overall effect: Z = 2.39 (P = 0.02)								
**1.6.2 Mepolizumab**								
Han 2021	1.7	10.8	54	0.4	8.6	54	20.8%	1.30 [-2.38, 4.98]
**Subtotal (95% CI)**			**54**			**54**	**20.8%**	**1.30 [-2.38, 4.98]**
Heterogeneity: Not applicable								
Test for overall effect: Z = 0.69 (P = 0.49)								
**Total (95% CI)**			**66**			**66**	**100.0%**	**2.09 [0.42, 3.77]**
Heterogeneity: Chi² = 0.22, df = 1 (P = 0.64); I² = 0%								
Test for overall effect: Z = 2.45 (P = 0.01)								
Test for subgroup differences: Chi² = 0.22, df = 1 (P = 0.64), I² = 0%								

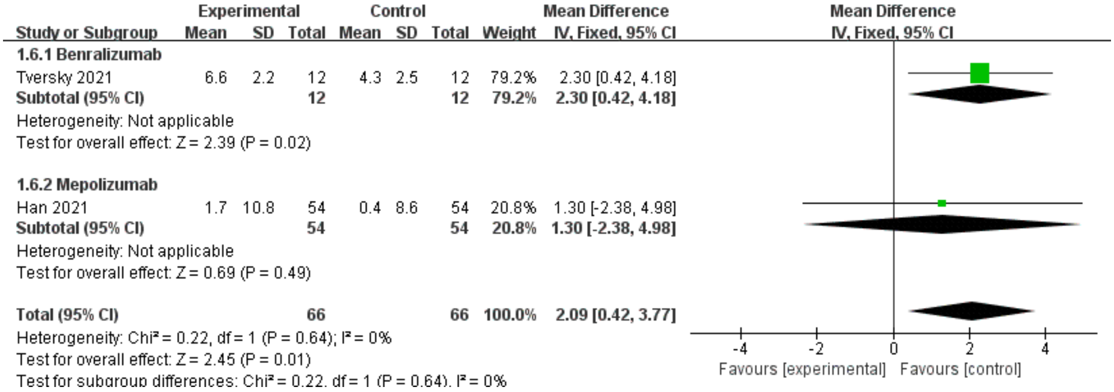

Figure S11. Forest plot of the meta-analysis for nasal overall VAS score

Study or Subgroup	Experimental Mean	Experimental SD	Experimental Total	Control Mean	Control SD	Control Total	Weight	Mean Difference IV, Fixed, 95% CI
**1.7.1 Mepolizumab**								
Fujieda 2024	-3.33	0.35	80	-1.8	0.35	83	97.2%	-1.53 [-1.64, -1.42]
Han 2021	-4.3	3.43	206	-2.5	3.08	201	2.8%	-1.80 [-2.43, -1.17]
**Subtotal (95% CI)**			**286**			**284**	**100.0%**	**-1.54 [-1.64, -1.43]**
Heterogeneity: Chi² = 0.68, df = 1 (P = 0.41); I² = 0%								
Test for overall effect: Z = 28.44 (P < 0.00001)								
**Total (95% CI)**			**286**			**284**	**100.0%**	**-1.54 [-1.64, -1.43]**
Heterogeneity: Chi² = 0.68, df = 1 (P = 0.41); I² = 0%								
Test for overall effect: Z = 28.44 (P < 0.00001)								
Test for subgroup differences: Not applicable								

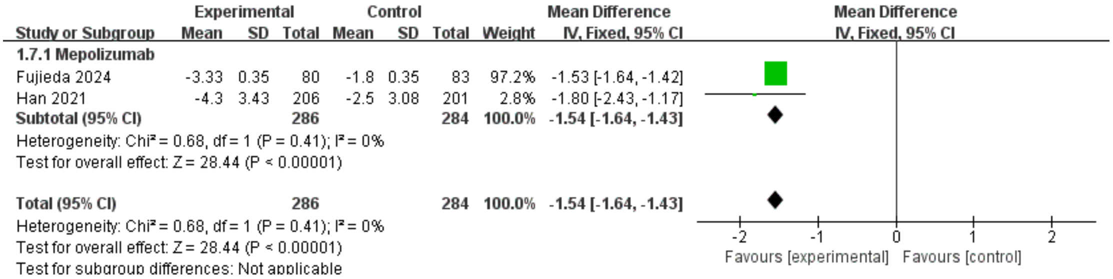

Figure S12. Forest plot of the meta-analysis for nasal composite VAS score

Study or Subgroup	Experimental Mean	Experimental SD	Experimental Total	Control Mean	Control SD	Control Total	Weight	Mean Difference IV, Random, 95% CI
**1.8.1 Mepolizumab**								
Fujieda 2024	-2.64	0.29	80	-1.47	0.29	83	73.5%	-1.17 [-1.26, -1.08]
Han 2021	-3.8	3.19	206	-2.2	2.82	201	26.5%	-1.60 [-2.18, -1.02]
**Subtotal (95% CI)**			**286**			**284**	**100.0%**	**-1.28 [-1.66, -0.91]**
Heterogeneity: Tau² = 0.05; Chi² = 2.03, df = 1 (P = 0.15); I² = 51%								
Test for overall effect: Z = 6.77 (P < 0.00001)								
**Total (95% CI)**			**286**			**284**	**100.0%**	**-1.28 [-1.66, -0.91]**
Heterogeneity: Tau² = 0.05; Chi² = 2.03, df = 1 (P = 0.15); I² = 51%								
Test for overall effect: Z = 6.77 (P < 0.00001)								
Test for subgroup differences: Not applicable								

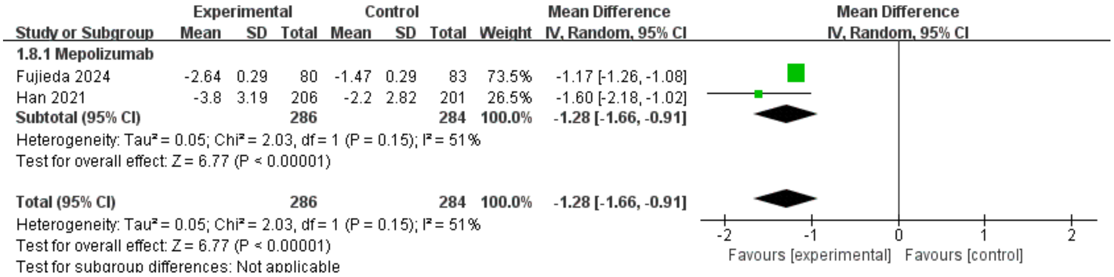

Figure S13. Forest plot of the meta-analysis for number of first-time NP surgery patients

Study or Subgroup	Experimental Events	Experimental Total	Control Events	Control Total	Weight	Risk Ratio M-H, Random, 95% CI
**2.14.1 benralizumab**						
Bachert 2022	33	207	37	203	33.4%	0.87 [0.57, 1.34]
**Subtotal (95% CI)**		**207**		**203**	**33.4%**	**0.87 [0.57, 1.34]**
Total events	33		37			
Heterogeneity: Not applicable						
Test for overall effect: Z = 0.61 (P = 0.54)						
**2.14.2 mepolizumab**						
Han 2021	18	206	46	201	29.6%	0.38 [0.23, 0.64]
**Subtotal (95% CI)**		**206**		**201**	**29.6%**	**0.38 [0.23, 0.64]**
Total events	18		46			
Heterogeneity: Not applicable						
Test for overall effect: Z = 3.71 (P = 0.0002)						
**2.14.3 depemokimab**						
Gevaert 2025	44	272	56	256	36.9%	0.74 [0.52, 1.06]
**Subtotal (95% CI)**		**272**		**256**	**36.9%**	**0.74 [0.52, 1.06]**
Total events	44		56			
Heterogeneity: Not applicable						
Test for overall effect: Z = 1.66 (P = 0.10)						
**Total (95% CI)**		**685**		**660**	**100.0%**	**0.64 [0.41, 1.00]**
Total events	95		139			
Heterogeneity: Tau² = 0.11; Chi² = 6.51, df = 2 (P = 0.04); I² = 69%						
Test for overall effect: Z = 1.94 (P = 0.05)						
Test for subgroup differences: Chi² = 6.48, df = 2 (P = 0.04), I² = 69.1%						

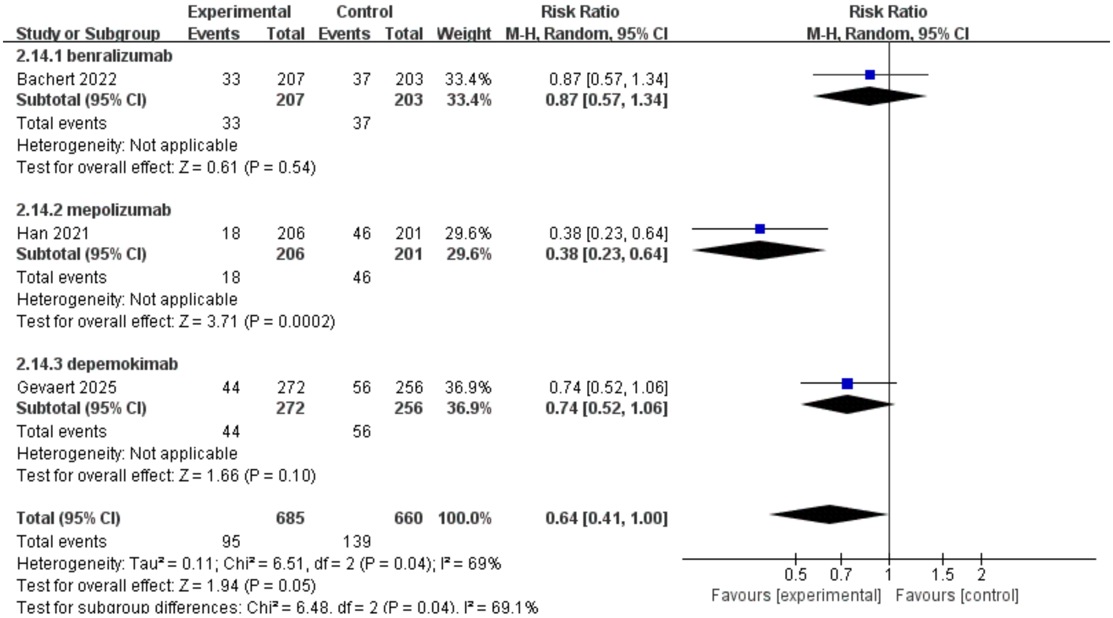

Figure S14. Forest plot of the meta-analysis for number of systemic corticosteroids (⩾1 course) patients

Study or Subgroup	Experimental Events	Experimental Total	Control Events	Control Total	Weight	Risk Ratio M-H, Fixed, 95% CI
**2.15.1 benralizumab**						
Bachert 2022	52	207	66	203	28.2%	0.77 [0.57, 1.05]
**Subtotal (95% CI)**		**207**		**203**	**28.2%**	**0.77 [0.57, 1.05]**
Total events	52		66			
Heterogeneity: Not applicable						
Test for overall effect: Z = 1.64 (P = 0.10)						
**2.15.2 mepolizumab**						
Han 2021	52	206	74	201	31.7%	0.69 [0.51, 0.92]
**Subtotal (95% CI)**		**206**		**201**	**31.7%**	**0.69 [0.51, 0.92]**
Total events	52		74			
Heterogeneity: Not applicable						
Test for overall effect: Z = 2.49 (P = 0.01)						
**2.15.3 depemokimab**						
Gevaert 2025	72	272	92	256	40.1%	0.74 [0.57, 0.95]
**Subtotal (95% CI)**		**272**		**256**	**40.1%**	**0.74 [0.57, 0.95]**
Total events	72		92			
Heterogeneity: Not applicable						
Test for overall effect: Z = 2.33 (P = 0.02)						
**Total (95% CI)**		**685**		**660**	**100.0%**	**0.73 [0.62, 0.86]**
Total events	176		232			
Heterogeneity: Chi² = 0.31, df = 2 (P = 0.86); I² = 0%						
Test for overall effect: Z = 3.75 (P = 0.0002)						
Test for subgroup differences: Chi² = 0.31, df = 2 (P = 0.86), I² = 0%						

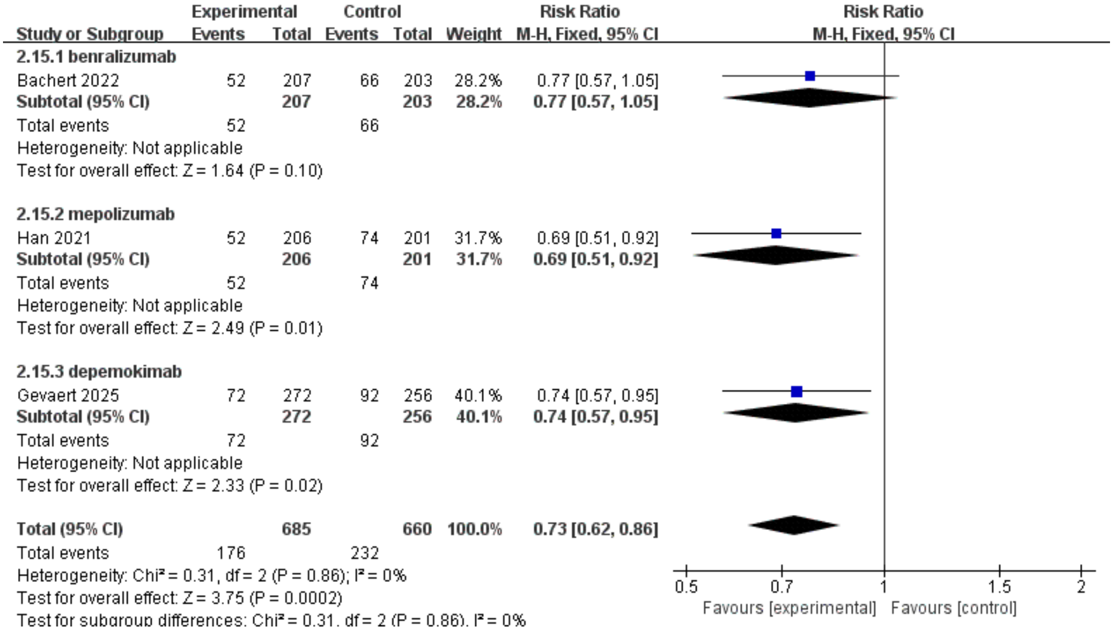

Figure S15. Forest plot of the meta-analysis for on-treatment AE (≥1 AE)

Study or Subgroup	Experimental Events	Experimental Total	Control Events	Control Total	Weight	Risk Ratio M-H, Fixed, 95% CI
Bachert 2017	40	53	42	52	6.1%	0.93 [0.76, 1.14]
Bachert 2022	160	207	160	203	23.1%	0.98 [0.89, 1.09]
Canonica 2022	73	96	42	57	7.5%	1.03 [0.85, 1.25]
Fujieda 2024	68	84	65	85	9.2%	1.06 [0.90, 1.24]
Gevaert 2006	7	8	4	4	0.8%	0.93 [0.61, 1.40]
Gevaert 2025	203	256	204	272	28.3%	1.06 [0.96, 1.16]
Han 2021	169	206	168	201	24.3%	0.98 [0.90, 1.07]
Takabayashi 2021	13	23	3	6	0.7%	1.13 [0.47, 2.72]
**Total (95% CI)**		**933**		**880**	**100.0%**	**1.01 [0.96, 1.06]**
Total events	733		688			

Heterogeneity: Chi² = 2.85, df = 7 (P = 0.90); I² = 0%
Test for overall effect: Z = 0.46 (P = 0.65)

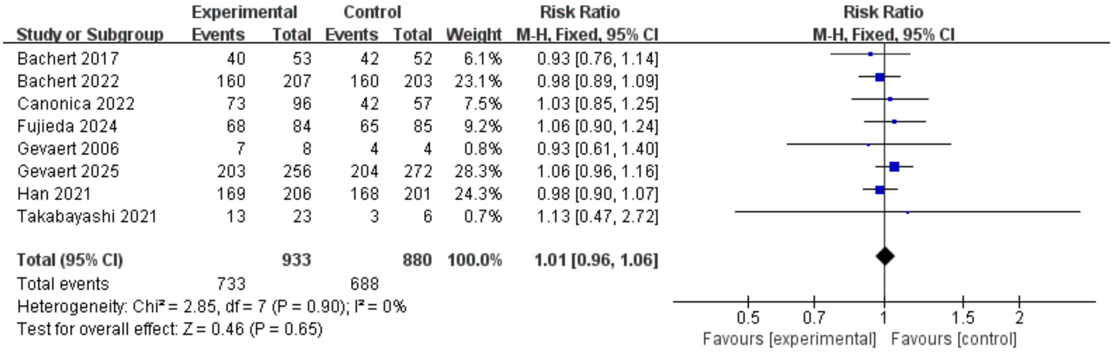

Figure S16. Figure S16. Forest plot of the meta-analysis for serious adverse events

Study or Subgroup	Experimental Events	Experimental Total	Control Events	Control Total	Weight	Risk Ratio M-H, Fixed, 95% CI
Bachert 2017	0	53	0	52		Not estimable
Bachert 2022	23	207	17	203	28.7%	1.33 [0.73, 2.41]
Canonica 2022	5	96	7	57	14.7%	0.42 [0.14, 1.27]
Fujieda 2024	0	84	4	85	7.5%	0.11 [0.01, 2.06]
Gevaert 2011	1	20	0	10	1.1%	1.57 [0.07, 35.46]
Gevaert 2025	11	256	16	272	26.0%	0.73 [0.35, 1.54]
Han 2021	12	206	13	201	22.0%	0.90 [0.42, 1.93]
**Total (95% CI)**		**922**		**880**	**100.0%**	**0.86 [0.60, 1.23]**
Total events	52		57			

Heterogeneity: Chi² = 5.85, df = 5 (P = 0.32); I² = 15%
Test for overall effect: Z = 0.84 (P = 0.40)

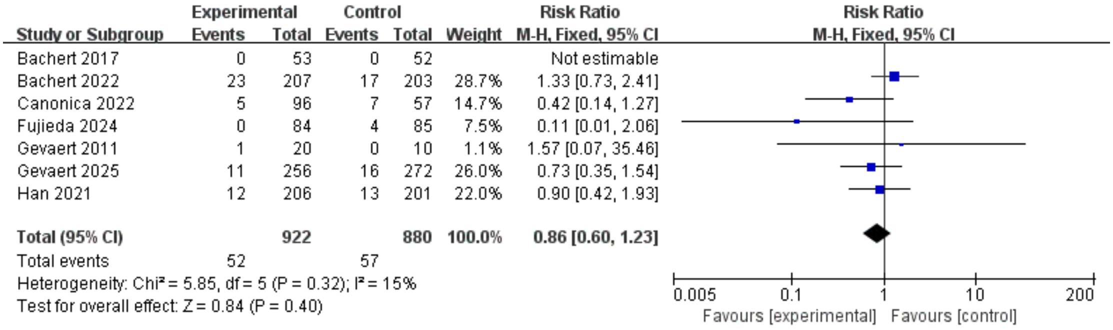

Figure S17. Forest plot of the meta-analysis for nasopharyngitis

Study or Subgroup	Experimental Events	Experimental Total	Control Events	Control Total	Weight	Risk Ratio M-H, Fixed, 95% CI
Bachert 2017	10	53	12	52	10.0%	0.82 [0.39, 1.73]
Bachert 2022	36	207	41	203	34.0%	0.86 [0.58, 1.29]
Canonica 2022	8	96	7	57	7.2%	0.68 [0.26, 1.77]
Fujieda 2024	7	84	9	85	7.4%	0.79 [0.31, 2.02]
Gevaert 2011	1	20	0	10	0.5%	1.57 [0.07, 35.46]
Han 2021	52	206	46	201	38.3%	1.10 [0.78, 1.56]
Takabayashi 2021	3	23	2	6	2.6%	0.39 [0.08, 1.84]
**Total (95% CI)**		**689**		**614**	**100.0%**	**0.92 [0.73, 1.16]**
Total events	117		117			

Heterogeneity: Chi² = 3.03, df = 6 (P = 0.80); I² = 0%
Test for overall effect: Z = 0.69 (P = 0.49)

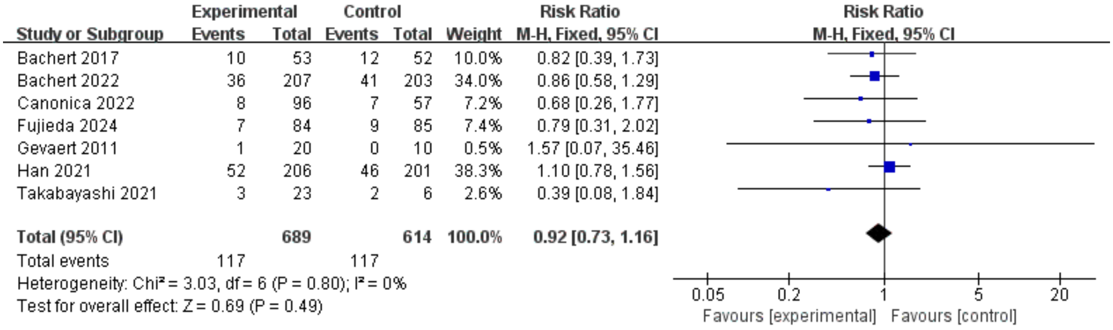

Figure S18. Forest plot of the meta-analysis for upper respiratory tract infection

Study or Subgroup	Experimental Events	Experimental Total	Control Events	Control Total	Weight	Risk Ratio M-H, Fixed, 95% CI
Bachert 2022	5	207	14	203	43.1%	0.35 [0.13, 0.95]
Gevaert 2006	5	8	2	4	8.1%	1.25 [0.41, 3.82]
Gevaert 2011	5	20	1	10	4.1%	2.50 [0.34, 18.63]
Han 2021	12	206	14	201	43.2%	0.84 [0.40, 1.76]
Takabayashi 2021	0	23	0	6		Not estimable
Tversky 2021	1	12	0	12	1.5%	3.00 [0.13, 67.06]
**Total (95% CI)**		**476**		**436**	**100.0%**	**0.76 [0.46, 1.25]**
Total events	28		31			

Heterogeneity: Chi² = 5.22, df = 4 (P = 0.27); I² = 23%
Test for overall effect: Z = 1.08 (P = 0.28)

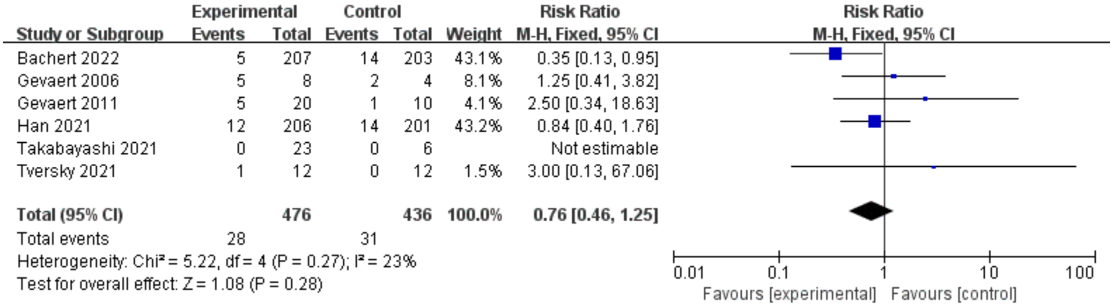

Figure S19. Forest plot of the meta-analysis for headache

Study or Subgroup	Experimental Events	Experimental Total	Control Events	Control Total	Weight	Risk Ratio M-H, Fixed, 95% CI
Bachert 2017	13	53	20	52	21.9%	0.64 [0.36, 1.14]
Bachert 2022	7	207	15	203	16.4%	0.46 [0.19, 1.10]
Canonica 2022	15	96	4	57	5.4%	2.23 [0.78, 6.38]
Fujieda 2024	6	84	6	85	6.5%	1.01 [0.34, 3.01]
Gevaert 2011	1	20	0	10	0.7%	1.57 [0.07, 35.46]
Han 2021	37	206	44	201	48.3%	0.82 [0.55, 1.21]
Takabayashi 2021	2	23	0	6	0.8%	1.46 [0.08, 26.97]
**Total (95% CI)**		**689**		**614**	**100.0%**	**0.82 [0.62, 1.08]**
Total events	81		89			

Heterogeneity: Chi^2 = 6.33, df = 6 (P = 0.39); I^2 = 5%
Test for overall effect: Z = 1.40 (P = 0.16)

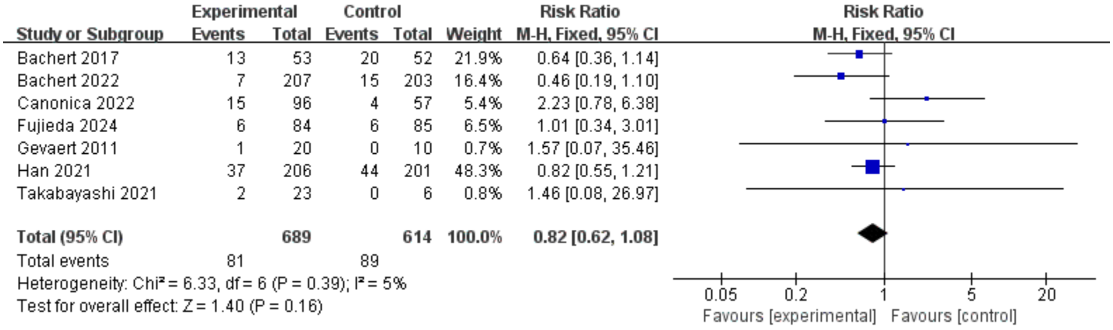

Figure S20. Forest plot of the meta-analysis for asthma

Study or Subgroup	Experimental Events	Experimental Total	Control Events	Control Total	Weight	Risk Ratio M-H, Fixed, 95% CI
Bachert 2017	2	53	3	52	5.4%	0.65 [0.11, 3.76]
Bachert 2022	19	207	29	203	52.0%	0.64 [0.37, 1.11]
Fujieda 2024	1	84	5	85	8.8%	0.20 [0.02, 1.70]
Han 2021	4	206	18	201	32.4%	0.22 [0.07, 0.63]
Takabayashi 2021	2	23	0	6	1.4%	1.46 [0.08, 26.97]
**Total (95% CI)**		**573**		**547**	**100.0%**	**0.48 [0.31, 0.74]**
Total events	28		55			

Heterogeneity: Chi^2 = 4.56, df = 4 (P = 0.34); I^2 = 12%
Test for overall effect: Z = 3.29 (P = 0.001)

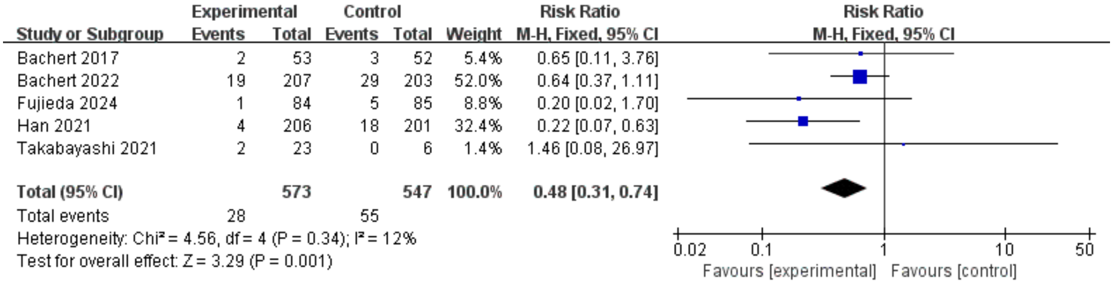

Figure S21. Forest plot of the meta-analysis for epistaxis

Study or Subgroup	Experimental Events	Experimental Total	Control Events	Control Total	Weight	Risk Ratio M-H, Fixed, 95% CI
Bachert 2017	1	53	3	52	14.3%	0.33 [0.04, 3.04]
Han 2021	17	206	18	201	85.7%	0.92 [0.49, 1.74]
**Total (95% CI)**		**259**		**253**	**100.0%**	**0.84 [0.46, 1.53]**
Total events	18		21			

Heterogeneity: Chi^2 = 0.77, df = 1 (P = 0.38); I^2 = 0%
Test for overall effect: Z = 0.58 (P = 0.56)

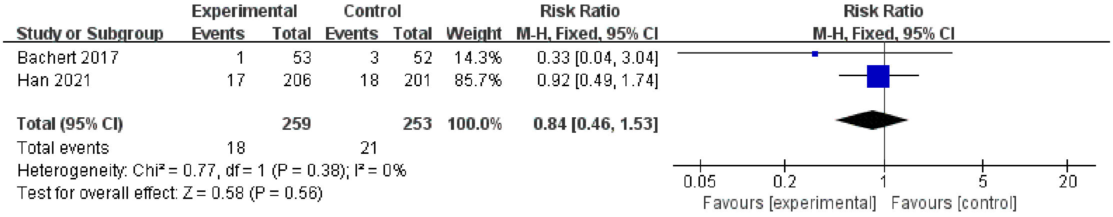

Figure S22. Forest plot of the meta-analysis for back pain

Study or Subgroup	Experimental Events	Experimental Total	Control Events	Control Total	Weight	Risk Ratio M-H, Fixed, 95% CI
Bachert 2017	5	53	0	52	2.6%	10.80 [0.61, 190.44]
Fujieda 2024	3	84	5	85	25.3%	0.61 [0.15, 2.46]
Han 2021	15	206	14	201	72.1%	1.05 [0.52, 2.11]
**Total (95% CI)**		**343**		**338**	**100.0%**	**1.19 [0.66, 2.12]**
Total events	23		19			

Heterogeneity: Chi^2 = 3.28, df = 2 (P = 0.19); I^2 = 39%
Test for overall effect: Z = 0.57 (P = 0.57)

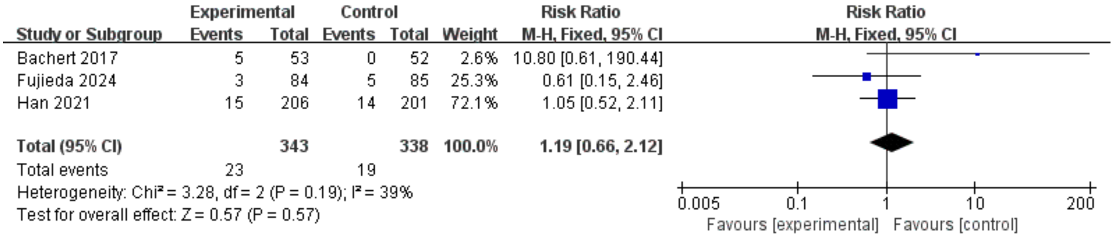

Figure S23. Forest plot of the meta-analysis for oropharyngeal pain

Study or Subgroup	Experimental Events	Experimental Total	Control Events	Control Total	Weight	Risk Ratio M-H, Fixed, 95% CI
Bachert 2017	6	53	4	52	28.5%	1.47 [0.44, 4.92]
Han 2021	16	206	10	201	71.5%	1.56 [0.73, 3.36]
**Total (95% CI)**		**259**		**253**	**100.0%**	**1.54 [0.80, 2.93]**
Total events	22		14			

Heterogeneity: Chi^2 = 0.01, df = 1 (P = 0.94); I^2 = 0%
Test for overall effect: Z = 1.30 (P = 0.19)

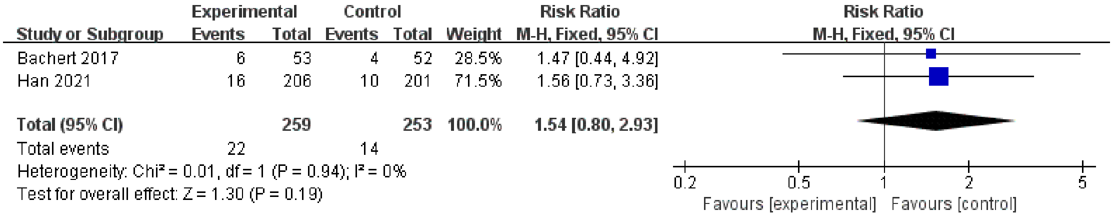

Figure S24. Forest plot of the meta-analysis for cough

Study or Subgroup	Experimental Events	Experimental Total	Control Events	Control Total	Weight	Risk Ratio M-H, Fixed, 95% CI
Bachert 2017	2	53	3	52	18.7%	0.65 [0.11, 3.76]
Han 2021	7	206	13	201	81.3%	0.53 [0.21, 1.29]
**Total (95% CI)**		**259**		**253**	**100.0%**	**0.55 [0.25, 1.22]**
Total events	9		16			

Heterogeneity: Chi² = 0.05, df = 1 (P = 0.83); I² = 0%
Test for overall effect: Z = 1.47 (P = 0.14)

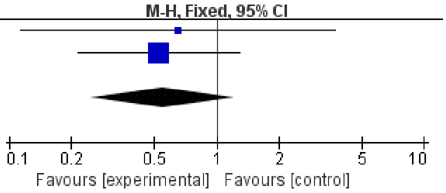

Figure S25. Forest plot of the meta-analysis for arthralgia

Study or Subgroup	Experimental Events	Experimental Total	Control Events	Control Total	Weight	Risk Ratio M-H, Fixed, 95% CI
Bachert 2017	3	53	3	52	37.4%	0.98 [0.21, 4.64]
Han 2021	13	206	5	201	62.6%	2.54 [0.92, 6.99]
**Total (95% CI)**		**259**		**253**	**100.0%**	**1.95 [0.85, 4.49]**
Total events	16		8			

Heterogeneity: Chi² = 1.01, df = 1 (P = 0.31); I² = 1%
Test for overall effect: Z = 1.58 (P = 0.11)

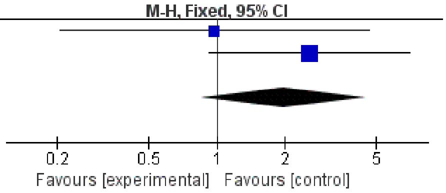

Figure S26. Forest plot of the meta-analysis for pyrexia

Study or Subgroup	Experimental Events	Experimental Total	Control Events	Control Total	Weight	Risk Ratio M-H, Fixed, 95% CI
Bachert 2017	3	53	1	52	9.8%	2.94 [0.32, 27.39]
Canonica 2022	7	96	3	57	36.4%	1.39 [0.37, 5.15]
Fujieda 2024	3	84	4	85	38.5%	0.76 [0.18, 3.29]
Takabayashi 2021	4	23	1	6	15.3%	1.04 [0.14, 7.70]
**Total (95% CI)**		**256**		**200**	**100.0%**	**1.24 [0.56, 2.77]**
Total events	17		9			

Heterogeneity: Chi² = 1.06, df = 3 (P = 0.79); I² = 0%
Test for overall effect: Z = 0.54 (P = 0.59)

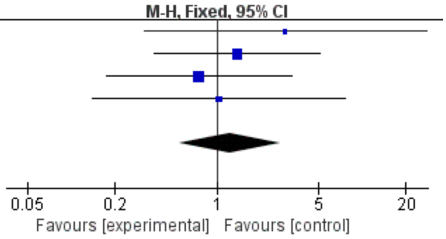

Figure S27. Forest plot of the meta-analysis for sinusitis

Study or Subgroup	Experimental Events	Experimental Total	Control Events	Control Total	Weight	Risk Ratio M-H, Fixed, 95% CI
Canonica 2022	9	96	2	57	9.4%	2.67 [0.60, 11.94]
Gevaert 2011	2	20	1	10	5.0%	1.00 [0.10, 9.75]
Han 2021	10	206	22	201	83.7%	0.44 [0.22, 0.91]
Tversky 2021	1	12	0	12	1.9%	3.00 [0.13, 67.06]
**Total (95% CI)**		**334**		**280**	**100.0%**	**0.73 [0.41, 1.29]**
Total events	22		25			

Heterogeneity: Chi² = 5.59, df = 3 (P = 0.13); I² = 46%
Test for overall effect: Z = 1.08 (P = 0.28)

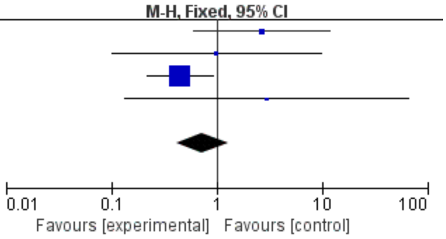

Figure S28. Forest plot of the meta-analysis for influenza

Study or Subgroup	Experimental Events	Experimental Total	Control Events	Control Total	Weight	Risk Ratio M-H, Random, 95% CI
Bachert 2017	4	53	2	52	45.2%	1.96 [0.38, 10.26]
Canonica 2022	5	96	0	57	28.5%	6.58 [0.37, 116.79]
Takabayashi 2021	0	23	1	6	26.3%	0.10 [0.00, 2.13]
**Total (95% CI)**		**172**		**115**	**100.0%**	**1.26 [0.16, 9.94]**
Total events	9		3			

Heterogeneity: Tau² = 1.75; Chi² = 4.17, df = 2 (P = 0.12); I² = 52%
Test for overall effect: Z = 0.22 (P = 0.83)

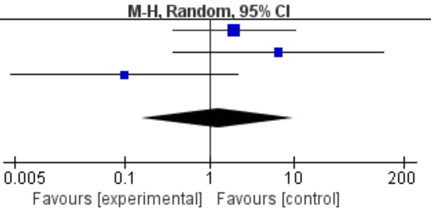

Supplement: Supplementary file 1 [file DataSheet1.pdf]
