# Supplementary material for: Safety and efficacy of anti-IL-5 monoclonal antibodies as second-line therapy for chronic rhinosinusitis with nasal polyps: a meta-analysis
Source: Front Immunol. 2026 Mar 30;17:1746573. doi: 10.3389/fimmu.2026.1746573 (PMC13070943; doi:10.3389/fimmu.2026.1746573)
Supplement: Supplementary file 2 [file DataSheet2.pdf]

PubMed		
No.	Query	Results
#1	anti-IL-5 monoclonal antibody[Title/Abstract] OR Antibodies, Monoclonal[Title/Abstract] OR Antibodies, Anti-Idiotypic[Title/Abstract] OR Antibodies, Monoclonal, Humanized[Title/Abstract] OR anti IL-5[Title/Abstract] OR Mepolizumab[Title/Abstract] OR SB-240563[Title/Abstract] OR SB240563[Title/Abstract] OR Nucala[Title/Abstract] OR Bosatria[Title/Abstract] OR Reslizumab[Title/Abstract] OR Cinqair[Title/Abstract] OR SCH-55700[Title/Abstract] OR SCH 55700[Title/Abstract] OR SCH55700[Title/Abstract] OR DCP-835[Title/Abstract] OR DCP 835[Title/Abstract] OR DCP835[Title/Abstract] OR CEP-38072[Title/Abstract] OR CEP38072[Title/Abstract] OR Benralizumab[Title/Abstract] OR MEDI-563[Title/Abstract] OR MEDI 563[Title/Abstract] OR Fasenra[Title/Abstract] OR BIW-8405[Title/Abstract] OR Depemokimab[Title/Abstract] OR SSGJ-610[Title/Abstract] OR SHR-1703[Title/Abstract] OR Varokibart[Title/Abstract] OR RC1416[Title/Abstract] OR PM1017[Title/Abstract] OR SY0828[Title/Abstract]	3,213
#2	rhinitis[Title/Abstract] OR Rhinitides[Title/Abstract] OR Nasal Catarrh[Title/Abstract] OR Catarrh, Nasal[Title/Abstract] OR Catarrhs, Nasal[Title/Abstract] OR Nasal Catarrhs[Title/Abstract] OR sinusitis[Title/Abstract] OR Sinusitides[Title/Abstract] OR Sinus Infections[Title/Abstract] OR Infection, Sinus[Title/Abstract] OR Infections, Sinus[Title/Abstract] OR Sinus Infection[Title/Abstract] OR rhinosinusitis[Title/Abstract] OR Rhino-sinusitis[Title/Abstract] OR Rhino sinusitis[Title/Abstract] OR nasal polyps[Title/Abstract] OR Nasal Polyp[Title/Abstract] OR Polyp, Nasal[Title/Abstract] OR Polyps, Nasal[Title/Abstract]	68,739
#3	(randomized controlled trial [pt] OR controlled clinical trial [pt] OR randomized [tiab] OR placebo [tiab] OR clinical trials as topic [mesh:noexp] OR randomly [tiab] OR trial [ti]) NOT (animals [mh] NOT humans [mh])	1,596,460
#4	#1 AND #2 AND #3	92

Web of Science		
No.	Query	Results
#1	TS=(anti-IL-5 monoclonal antibody OR Antibodies, Monoclonal OR Antibodies, Anti-Idiotypic OR Antibodies, Monoclonal, Humanized OR anti IL-5 OR Mepolizumab OR SB-240563 OR SB240563 OR Nucala OR Bosatria OR Reslizumab OR Cinqair OR SCH-55700 OR SCH 55700 OR SCH55700 OR DCP-835 OR DCP 835 OR DCP835 OR CEP-38072 OR CEP38072 OR Benralizumab OR MEDI-563 OR MEDI 563 OR Fasenra OR BIW-8405 OR Depemokimab OR SSGJ-610 OR SHR-1703 OR Varokibart OR RC1416 OR PM1017 OR SY0828)	306,970
#2	TS=(rhinitis OR Rhinitides OR Nasal Catarrh OR Catarrh, Nasal OR Catarrhs, Nasal OR Nasal Catarrhs OR sinusitis OR Sinusitides OR Sinus Infections OR	76,540

	Infection, Sinus OR Infections, Sinus OR Sinus Infection OR rhinosinusitis OR Rhino-sinusitis OR Rhino sinusitis OR nasal polyps OR Nasal Polyp OR Polyp, Nasal OR Polyps, Nasal)	
#3	TS=(randomized controlled trial OR controlled clinical trial OR randomized OR placebo OR clinical trials as topic OR randomly OR trial)	3,029,806
#4	#1 AND #2 AND #3	573

Embase		
No.	Query	Results
#1	'anti-il-5 monoclonal antibody':ti,ab,kw OR 'antibodies,monoclonal':ti,ab,kw OR 'antibodies,anti-idiotypic':ti,ab,kw OR 'antibodies,monoclonal,humanized':ti,ab,kw OR 'anti il-5':ti,ab,kw OR 'mepolizumab':ti,ab,kw OR 'sb-240563':ti,ab,kw OR 'sb240563':ti,ab,kw OR 'nucala':ti,ab,kw OR 'bosatria':ti,ab,kw OR 'reslizumab':ti,ab,kw OR 'cinqair':ti,ab,kw OR 'sch-55700':ti,ab,kw OR 'sch 55700':ti,ab,kw OR 'sch55700':ti,ab,kw OR 'dcp-835':ti,ab,kw OR 'dcp 835':ti,ab,kw OR 'dcp835':ti,ab,kw OR 'cep-38072':ti,ab,kw OR 'cep38072':ti,ab,kw OR 'benralizumab':ti,ab,kw OR 'medi-563':ti,ab,kw OR 'medi 563':ti,ab,kw OR 'fasenra':ti,ab,kw OR 'biw-8405':ti,ab,kw OR 'depemokimab':ti,ab,kw OR 'ssgj-610':ti,ab,kw OR 'shr-1703':ti,ab,kw OR 'varokibart':ti,ab,kw OR 'rc1416':ti,ab,kw OR 'pm1017':ti,ab,kw OR 'sy0828':ti,ab,kw	6,508
#2	'rhinitis':ti,ab,kw OR 'rhinitides':ti,ab,kw OR 'nasal catarrh':ti,ab,kw OR 'catarrh, nasal':ti,ab,kw OR 'catarrhs, nasal':ti,ab,kw OR 'nasal catarrhs':ti,ab,kw OR 'sinusitis':ti,ab,kw OR 'sinusitides':ti,ab,kw OR 'sinus infections':ti,ab,kw OR 'infection, sinus':ti,ab,kw OR 'infections, sinus':ti,ab,kw OR 'sinus infection':ti,ab,kw OR 'rhinosinusitis':ti,ab,kw OR 'rhino-sinusitis':ti,ab,kw OR 'rhino sinusitis':ti,ab,kw OR 'nasal polyps':ti,ab,kw OR 'nasal polyp':ti,ab,kw OR 'polyp, nasal':ti,ab,kw OR 'polyps, nasal':ti,ab,kw	98,816
#3	'randomized controlled trial'/exp OR 'randomized controlled trial':ti,ab,it OR 'randomized':ti,ab,it OR 'randomised':ti,ab,it OR 'randomization':ti,ab,it OR 'randomisa- tion':ti,ab,it OR rct:ti,ab,it OR 'randomly':ti,ab,it OR pla-cebo:ti,ab,it	2,213,436
#4	#1 AND #2 AND #3	169

cochrane library		
No.	Query	Results
#1	(anti-IL-5 monoclonal antibody OR Antibodies,Monoclonal OR Antibodies,Anti-Idiotypic OR Antibodies,Monoclonal,Humanized OR anti IL-5 OR Mepolizumab OR SB-240563 OR SB240563 OR Nucala OR Bosatria OR Reslizumab OR Cinqair OR SCH-55700 OR SCH 55700 OR SCH55700 OR DCP-835 OR DCP 835 OR DCP835 OR CEP-38072 OR CEP38072 OR Benralizumab OR MEDI-563 OR MEDI 563 OR Fasenra OR BIW-8405):ti,ab,kw	17245
#2	(rhinitis OR Rhinitides OR Nasal Catarrh OR Catarrh, Nasal OR Catarrhs, Nasal OR Nasal Catarrhs OR sinusitis OR Sinusitides OR Sinus Infections OR Infection,	16416

	Sinus OR Infections, Sinus OR Sinus Infection OR rhinosinusitis OR Rhino-sinusitis OR Rhino sinusitis OR nasal polyps OR Nasal Polyp OR Polyp, Nasal OR Polyps, Nasal):ti,ab,kw	
#3	(randomized controlled trial OR controlled clinical trial OR randomized OR placebo OR clinical trials as topic OR randomly OR trial):ti,ab,kw	1624967
#4	#1 AND #2 AND #3	481
